# Robustness of radiomic features in CT images with different slice thickness, comparing liver tumour and muscle

**DOI:** 10.1038/s41598-021-87598-w

**Published:** 2021-04-15

**Authors:** Lorena Escudero Sanchez, Leonardo Rundo, Andrew B. Gill, Matthew Hoare, Eva Mendes Serrao, Evis Sala

**Affiliations:** 1 Department of Radiology, University of Cambridge, Cambridge, CB2 0QQ UK; 2 Cancer Research UK Cambridge Centre, University of Cambridge, Cambridge, UK; 3 Cambridge University Hospitals NHS Foundation Trust, Addenbrooke’s Hospital, Cambridge, UK; 4 Department of Medicine, University of Cambridge, Cambridge, UK

**Keywords:** Cancer imaging, Tumour biomarkers, Tumour heterogeneity, Liver cancer, Liver cancer, Cancer imaging, Tumour biomarkers, Tumour heterogeneity, Computational science, Biomedical engineering

## Abstract

Radiomic image features are becoming a promising non-invasive method to obtain quantitative measurements for tumour classification and therapy response assessment in oncological research. However, despite its increasingly established application, there is a need for standardisation criteria and further validation of feature robustness with respect to imaging acquisition parameters. In this paper, the robustness of radiomic features extracted from computed tomography (CT) images is evaluated for liver tumour and muscle, comparing the values of the features in images reconstructed with two different slice thicknesses of 2.0 mm and 5.0 mm. Novel approaches are presented to address the intrinsic dependencies of texture radiomic features, choosing the optimal number of grey levels and correcting for the dependency on volume. With the optimal values and corrections, feature values are compared across thicknesses to identify reproducible features. Normalisation using muscle regions is also described as an alternative approach. With either method, a large fraction of features (75–90%) was found to be highly robust (< 25% difference). The analyses were performed on a homogeneous CT dataset of 43 patients with hepatocellular carcinoma, and consistent results were obtained for both tumour and muscle tissue. Finally, recommended guidelines are included for radiomic studies using variable slice thickness.

## Introduction

Hepatocellular carcinoma (HCC) is the most common liver primary tumour, and currently the 4th largest cause of cancer-related death^1,2^ with a poor survival rate (less than 20% 5-year survival rate^3^). Radiomics analysis of HCC cases has been used to predict outcome^4^ and early recurrence^5^ using computed tomography (CT) images, as well as defining tumour immunoscore^6^ based on Magnetic Resonance Imaging (MRI). However, despite these advances, the reported radiomics quality score (RQS)^7^ of HCC radiomic studies is low ($8.35\pm 5.38$ out of a possible maximum value of 36)^8^. Therefore, careful analyses of radiomic feature robustness and reliability require attention from the scientific community.

Radiomics^9^, the emerging field of research describing the extraction of mineable features from medical images, is based on the idea that medical images convey information that reflects the underlying pathophysiology, which can be probed by quantitative image analysis. Unlike qualitative image evaluation, which requires a trained reader to make a subjective judgement based on images (i.e. presence of disease), radiomics allows a large number of quantitative features to be extracted from standard-of-care images, from modalities such as CT, MRI and Positron Emission Tomography (PET). These features provide quantitative measurements of tissue characteristics, such as shape or heterogeneity, allowing objective, reader-independent non-invasive biomarkers to be extracted.

Radiomics has shown great potential in precision oncology^10^, but there is still a need for safeguards and standardisation to achieve robust and generalisable results^11^. Efforts to harmonise the study of radiomic features are being made within the cancer research community, such as those provided by the Image Biomarker Standardisation Initiative (IBSI)^12^. However, these attempts are not yet offering comprehensive guidelines in order to make practical choices—such as decisions on voxel size and quantisation of grey levels (GLs), necessary to obtain robust and reliable outcomes^13^.

First of all, it is necessary to assess feature robustness in terms of the intrinsic dependencies of some features on volume (or number of voxels) and on the number of GLs^14^, regardless of the software package used to calculate these features^13^. This paper presents novel approaches to address such intrinsic dependencies, with the aim to choose the optimal number of GLs and voxel size. The analyses performed focus on evaluating the impact of such choices on images with different reconstructed slice thicknesses—which is one of the main acquisition parameters that varies in realistic cohorts—by comparing the values obtained using images reconstructed with 2.0 mm and 5.0 mm, typical slice thickness values used in diagnostic CT scans. An automatic, software-agnostic method to correct for volume dependencies of some of the texture features is also presented.

Once the optimal choice for GLs has been identified, and the optimal correction of dependencies on the number of voxels has been implemented, the values of the features are compared for the different slice thicknesses in order to (1) choose the optimal voxel size for the feature extraction and (2) quantify the number of features that are robust with respect to a change of slice thickness. Results are presented for liver tumour and muscle, with consistent conclusions extracted for both tissue types.

The present work is, to the best of our knowledge, the first study that systematically investigates radiomic feature robustness for the same CT scans reconstructed with two different slice thickness values. In addition to the methods described to correct for intrinsic dependencies of the parameters and minimise the effect of the slice thickness, recommended guidelines are provided to make optimal choices in future radiomic studies analysing heterogeneous cohorts with variable slice thickness.

## Materials and methods

### Dataset composition and preparation

This retrospective analysis was conducted on imaging from patients on the liver transplant waiting list. Patients were recruited at the Cambridge University Hospitals NHS Foundation Trust, Cambridge, UK, and gave written informed consent with approval of the Office for Research Ethics Committees Northern Ireland (REC ref 16/NI/0196, IRAS 206106, REC approval on September 12th 2016). After subsequent transplantation all were demonstrated to have histologically-proven HCC. All transplants conducted between 2002 and 2020 were retrospectively reviewed, and a total of $n=43$ were selected between the years 2005–2020. To protect the individuals’ privacy, the patient’s exam information was pseudo-anonymised by replacing personal identifiers with pseudonyms. All work was carried out in accordance with relevant guidelines and regulations.

Patients were selected based on the criterion that contrast-enhanced CT scans were available with homogeneous CT acquisition parameters (except for in-plane resolution and slice thickness, focus of our analysis) and that images were obtained as part of the same study with two reconstructed slice thickness of 2.0 mm and 5.0 mm. The CT scans were all acquired at porto-venous contrast phase and a 120 kilovoltage peak (KVP) and they were reconstructed with a convolution kernel of either B20f or B30f (all B20f for 2.0 mm). The pixel spacing (i.e. in-plane resolution) varied within a range of (0.57–0.92) mm, with a mean of $\mu _{x} = \mu _{y} = 0.735$ mm. The relevant CT acquisition parameters are summarised in Supplementary Table ST1. The main demographics characteristics of the selected $n=43$ patients are summarised in Supplementary Table ST2.

A radiologist (E.S.M.) with 6 years of experience segmented the ROIs for the liver tumours. A single tumour was delineated per patient and analysed. In addition, ROIs were also drawn in the CT images within healthy muscle tissue, in the right erector spinae, such that every tumour ROI was accompanied on the same slice by an ROI of similar size drawn within the muscle, resulting in comparable 3D volumes for the tumour and muscle. All scans and ROIs were delineated using the Microsoft Radiomics Tool (Version 1.0.30558.1, project InnerEye^15^, Redmond, WA, USA). The segmentation of liver tumours and muscle regions was performed for each patient and study in scans with 2.0 mm and 5.0 mm slice thickness. Potential volume effects or biases in the segmentation of the tumours, as a function of the slice thickness used, were investigated with the Bland–Altman analysis^16^.

### Radiomic features extraction and pre-processing

The radiomic features considered in this study were computed using PyRadiomics (version 2.2.0)^17^, an open source Python package widely used for this purpose. Since this software requires the image input in the Neuroimaging Informatics Technology Initiative (NIfTI) format^18^, a preliminary step was performed to convert the original Digital Imaging and Communications in Medicine (DICOM) scan and segmentation files to this format using custom software written in MATLAB (The Mathworks Inc., Natick, MA, USA) version R2019b.

A total of 107 3D radiomic features were calculated, without any image filters applied, from the following categories: *shape* (14), *first-order* (18), Grey Level Co-occurrence Matrix features (*GLCM*)^19–21^ (24), Grey Level Dependence Matrix (*GLDM*)^22^ (14), Grey Level Run Length Matrix (*GLRLM*)^23^ (16), Grey Level Size Zone Matrix (*GLSZM*)^24^ (16) and Neighbouring Grey Tone Difference Matrix Features (*NGTDM*)^25^ (5). The full list of features can be found in Supplementary Tables ST6-ST7.

The analyses described in this paper were performed using the ROOT^26^ open-source data analysis framework developed at CERN (Geneva, Switzerland). This was used *via* its Python interface to integrate it with other Python libraries, such as NumPy, SciPy and scikit-learn^27^.

### Intrinsic dependencies of radiomic features

In this paper, the known intrinsic dependencies of some of the radiomic image features studied are: (1) the number of GLs used—also called quantisation—and (2) the outlined (tumour) volume, expressed as number of voxels.

#### Choice of grey level quantisation

A number of GLs, resulting from a process known as quantisation or re-binning, needs to be selected in order to extract radiomic features. This number typically has an impact on the distribution of intensity values (i.e. histogram) of the volume considered, and on the GL matrices that are calculated comparing local image intensities, such as co-occurrence (*GLCM*) and run-length (*GLRLM*) matrices. Thus, the chosen number of GLs might have an effect on the values of certain radiomic features.

Two options are typically available for choosing the number of GLs: either fixing the number of bins, or fixing the bin width. Although fixing the bin width seems naturally more appropriate when comparing histograms of different extent, the IBSI guidelines^12^ recommend using a fixed number of bins instead. The data used in this study have the advantage of very little variation in the image intensity ranges across the different patients. In this case using a fixed number of bins or a fixed bin width become equivalent. Therefore, a fixed number of bins was preferred in this analysis.

In order to test the effect of the GL quantisation, and to evaluate its impact on the values of the radiomic features, seeking to find the optimal choice minimising differences in a cohort of heterogeneous slice thickness, radiomic features were extracted using different values of number of GLs, following powers of two as they are typically used in the literature: 8, 16, 32, 64, 128 and 256. In addition, a statistical rule, known as the Freedman–Diaconis rule^28^, was explored to calculate the optimal GL number automatically. This rule is a basic extension, generalised to non-Gaussian distributions, of Scott’s rule^29^, which is a statistical attempt to find the optimal bin width of a distribution (histogram) for an unbiased estimation of the probability density function underneath. The Freedman–Diaconis rule is based on the interquartile range (IQR) and states that the optimal bin width of a distribution $\mathsf {X}$ can be computed as:

1 $$\begin{aligned} \text {width}_{\text {bins}} = 2 \frac{\text {IQR}(\mathsf {X})}{N^{1/3}}, \end{aligned}$$

where *N* is the number of entries (i.e. elements) in the distribution $\mathsf {X}$, and that can be translated as:

2 $$\begin{aligned} n_{\text {bins}} = \frac{\mathsf {X}_{\max }-\mathsf {X}_{\min }}{\text {width}_{\text {bins}}} = \frac{1}{2}\frac{(\mathsf {X}_{\max }-\mathsf {X}_{\min })N^{1/3}}{\text {IQR}(\mathsf {X})}. \end{aligned}$$

The intraclass correlation coefficient (ICC) was computed to evaluate which features are correlated with the number of GLs. Given *k* multiple measurements to be compared (7 different GL quantisations), $\text {ICC}(3,1)$^30^ for a two-way random-effects (or mixed effects) model was used:

3 $$\begin{aligned} \text {ICC}(3,1) = \frac{\text {MS}_{R} - \text {MS}_{E}}{\text {MS}_{R} + (k-1) \text {MS}_{E}}, \end{aligned}$$

where $\text {MS}_R$ and $\text {MS}_E$ are the mean square for rows and mean square for error, respectively.

#### Correction of volume dependencies

The other known key dependency of some radiomic features is the volume of the ROI considered. This dependency was analysed as a function of the number of voxels.

There are two ways in which the number of voxels can be varied: (a) by fixing the voxel size, and analysing ROIs with different 3D volumes, and (b) for a fixed 3D volume (ROI), varying the size of the voxels used in the calculation. In this study, both variations were considered (a) by collecting data from different patients, for which ROI volumes differ; and (b) by varying the voxel sizes, both by resampling the images within patients and by comparing the studies with 2.0 mm and 5.0 mm slice thickness.

The images were resampled using the following set of isotropic and anisotropic voxel sizes. This set includes some standard choices found in the literature and ensures that examples of both up-sampling and down-sampling are considered:

- Original, i.e. no resampling;
- Isotropic choices; (0.5, 0.5, 0.5) mm, (1.0, 1.0, 1.0) mm, (1.25, 1.25, 1.25) mm, (1.75, 1.75, 1.75) mm and (2.0, 2.0, 2.0) mm;
- ($\mu _x$, $\mu _y$, Z) where $\mu _{x}=\mu _{y}=0.735$ mm is the mean of the pixel widths and Z is the original thickness (2.0 mm or 5.0 mm);
- ($\mu _{x}$, $\mu _{y}$, $\mu _{z}$) where $\mu _{x}=\mu _{y}=0.735$ mm is the mean of the pixel widths and $\mu _{z}=3.5$ mm is the mean thickness.

For each resampling, three different interpolation methods were compared: linear, B-spline (spline) and Welch windowed sinc interpolator (welch).

Features that show a dependency on the number of voxels were identified by evaluating the Spearman’s rank correlation coefficient ($r_S$), deeming them as correlated if $r_{S}>=0.5$.

A correction of the volume dependency was explored for those features correlated. The purpose of this correction is not to discard any valuable information that can be extracted from the volume itself, therefore it was not applied to shape-related features. Instead, the aim is to decouple from volume effects the potential predictive power of radiomic features that might be complementary to the information conveyed by the volume itself.

For this purpose, an automatic approach was developed to perform fits to estimate the underlying function that describes the correlation for each feature: $y = f(x)$ where *y* is each radiomic feature and *x* is the number of voxels. Different invertible functions were tested for *f*(*x*), covering the dependencies reported in the literature^9^ and beyond. Therefore, eight fits were performed per feature with the following formulae, each of them described by two coefficients or fit parameters [*p*0] and [*p*1]: (1) $f(x) = [p1] \cdot x + [p0]$; (2) $f(x) = [p1] \cdot x^2 + [p0]$; (3) $f(x) = [p1]\cdot x^3 + [p0]$; (4) $f(x) = [p1]/x + [p0]$; (5) $f(x) = [p1]/x^2 + [p0]$; (6) $f(x) = [p1]/x^3 + [p0]$; (7) $f(x) = [p1] \cdot \log (x) + [p0]$; (8) $f(x) = [p1]/\log (x) + [p0]$. From these, the function that best fits the distribution of each radiomic feature versus the number of voxels was the one adopted to correct for its observed dependency. In this way, this approach was not only automatic but also software-agnostic and did not need any prior knowledge of the feature derivation, working for any feature extraction platform^13^.

A necessary intermediate step to achieve reliable fits was to combine the feature measurements (one per patient) together to compute an estimation of the statistical uncertainty on the measurements. To do so, feature measurements with similar numbers of voxels were combined into a single point (mean of the measurements) and error (standard deviation). To ensure an approximately constant number of measurements (and at least 2 in all cases) is used for each point, divisions of variable width are used for the number of voxels (X axis). This uncertainty could then be used in the fit to evaluate a meaningful metric $\chi ^2$, which allowed for poor fits to be discarded and to select the function best fitting the distribution of data points. This step is illustrated in Fig. 4 (middle row).

### Robustness of radiomic features in different slice thicknesses

For the comparison of feature values in different slice thicknesses, the main metric used in this study was the relative difference between the values of feature *f* in 2.0 mm and 5.0 mm as:

4 $$\begin{aligned} \Delta _{r}=\frac{|f_{5\,\text {mm}}-f_{2\,\text {mm}}|}{|f_{2\,\text {mm}}|}. \end{aligned}$$

In addition to $\Delta _r$, the variance of the values of the features within patients was also taken into account. This was measured by the normalised standard deviation:

5 $$\begin{aligned} \sigma _{n}=\frac{\sigma (f)}{|\text {median}(f)|}, \end{aligned}$$

where $\sigma$ is the standard deviation of *f*, which is the value of the feature within the cohort of patients. This additional metric was used to establish which features presented a very small (or near-zero) variance in certain scenarios (e.g. with a given number of GLs tested).

### Normalisation using healthy muscle tissue

As an additional test to improve the robustness of the texture radiomic features (i.e. all except those relating to shape), normalisation with respect to the healthy muscle tissue obtained from the muscle ROIs was investigated. To that effect, ratios of the values of the features were obtained as:

6 $$\begin{aligned} \rho =\frac{f_{\text {tumour}}}{f_{\text {muscle}}}, \end{aligned}$$

and they were used to calculate the corresponding metric showing the difference between those ratios for images with 2.0 mm and 5.0 mm slice thickness according to Eq. (6):

7 $$\begin{aligned} \Delta _{\rho }=\frac{|\rho _{5\,\text {mm}}-\rho _{2\,\text {mm}}|}{|\rho _{2\,\text {mm}}|}. \end{aligned}$$

## Results

**Figure 1:**
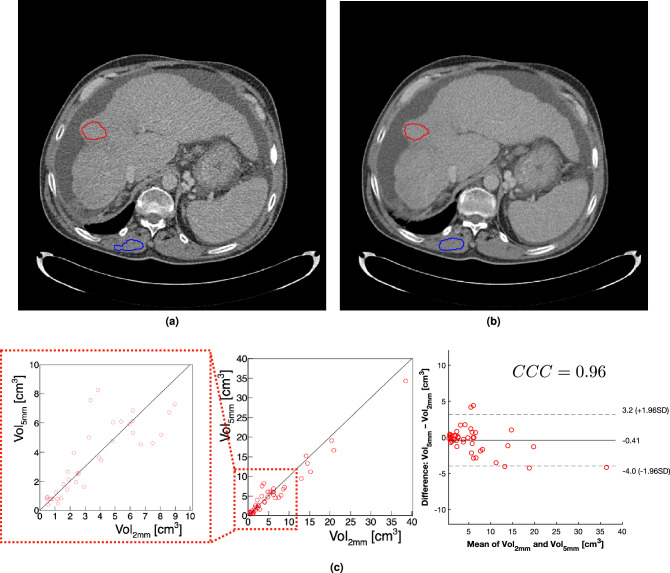
Example of the segmentation performed in a 2D slice for one patient in CT images acquired with 2.0 mm (**a**) and 5.0 mm (**b**) slice thickness, showing the HCC tumour (red) and muscle (blue) regions of interest. Comparison of liver tumour volumes (**c**) calculated using the segmented CT scans with 2.0 mm (Vol$_{2\,\text {mm}}$) and 5.0 mm (Vol$_{5\,\text {mm}}$) slice thickness (middle), showing a zoom of the Vol $< 10 \,\text {cm}^3$ region (left). The comparison of these measurements is summarised in the Bland–Altman analysis (right), with the Lin’s concordance correlation coefficient (CCC) showing a high level of agreement between both measurements.

For each patient, the tumour and muscle ROIs were drawn in the two sets of acquired images, with 2.0 mm and 5.0 mm slice thicknesses. An example illustrating the segmentation performed for one patient in a 2D slice is shown in Fig.1 for 2.0 mm (Fig. 1a) and 5.0 mm (Fig. 1b).

The ranges of volumes were (calculated from 2.0 mm slice thickness images for reference):

- Tumour: approx. (0.4–38) cm$^3$, which corresponds to a range of (300–53141) number of voxels (original voxel sizes);
- Muscle: approx. (0.4–27) cm$^3$, which corresponds to a range of (304–37294) number of voxels (original voxel sizes).

The comparison of the volumes with 2.0 mm and 5.0 mm and the Bland–Altman analysis are presented in Fig. 1c, and show that, with the exception of the few outlier cases with very large volumes, there is no systematic bias in volume when comparing the results from both slice thicknesses.

### Intrinsic dependencies of radiomic features

Most of the radiomic features were found to have a correlation or dependency on either the number of GLs or the number of voxels. Some features presented both dependencies. In this section, results evaluating those intrinsic dependencies are discussed for the texture radiomic features: *first-order*, *GLCM*, *GLDM*, *GLRLM*, *GLSZM*, *NGTDM*.

#### Choice of grey level quantisation

**Figure 2:**
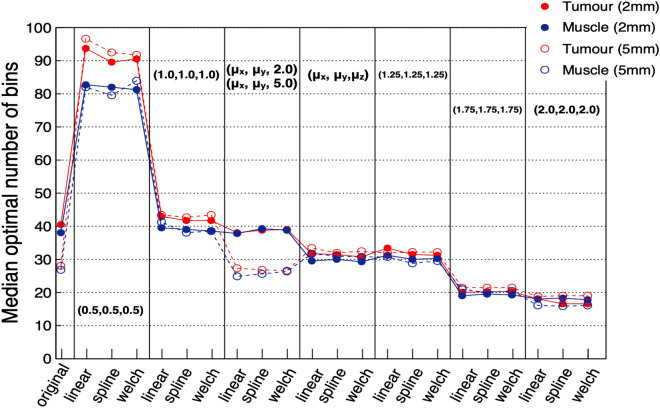
Result of the Freedman–Diaconis rule to calculate the optimal number of bins describing the distribution of pixel intensities of the ROIs for tumour (red) and muscle (blue). Results are presented for all voxel sizes and interpolators tested, providing the median across all patients. Values of $\mu _{x,y,z}$ represent the mean of the voxel widths of the original images in the corresponding *x*, *y*, *z* axis.

Figure 2 shows the result of the Freedman–Diaconis rule from Eq. (2). Note that the number of entries *N* in this case is the number of voxels and therefore depends on the voxel size used (listed in “Materials and methods”). The value obtained from this rule is $\sim$ 30–40 bins for most voxel sizes, however it significantly changed for very small or very large voxel sizes (maximum $\sim \,90$ bins, minimum $\sim\, 20$ bins). Values were also consistent between tumour and muscle, except for voxel size (0.5, 0.5, 0.5) mm.

Different grey level quantisation configurations (as listed in Materials and Methods) were compared, and the values of the features computed with each choice can be found in Supplementary Figures SF1-SF3, clearly showing a dependency of some features with the number of GLs. To minimise effects arising from using different number of voxels, which are confounded by the effect of change of GL bins, the 3D volumes were resampled to a common isotropic voxel size of (1,1,1) mm using the default interpolator in PyRadiomics (i.e. spline). For this voxel size, the corresponding value from the Freedman–Diaconis rule was 40 bins, which was then added to the set of analysed GLs (as per Materials and Methods).

Features with ICC(3,1) $<0.9$ were considered unstable and therefore significantly correlated with the number of GLs. A total of 70 features were found to be correlated with the number of GLs, and their ICC values (calculated independently for 2.0 mm and 5.0 mm, tumour and muscle) are presented in Supplementary Tables ST3 and ST4. This shows that values were in general consistent between both tissue types and between both slice thicknesses.

For those features correlated with the number of GLs, a comparison between the values with 2.0 mm and 5.0 mm calculated using Eq. (4) was performed to test the effect of the GL quantisation on the feature robustness. In addition, the variance within patients was also evaluated according to Eq. (5), and it was found that some features clearly presented a very small (or near-zero) variance when using extreme values of GLs (e.g. 8 or 256 bins), as can be observed in Supplementary Figures SF1-SF3.

**Figure 3:**
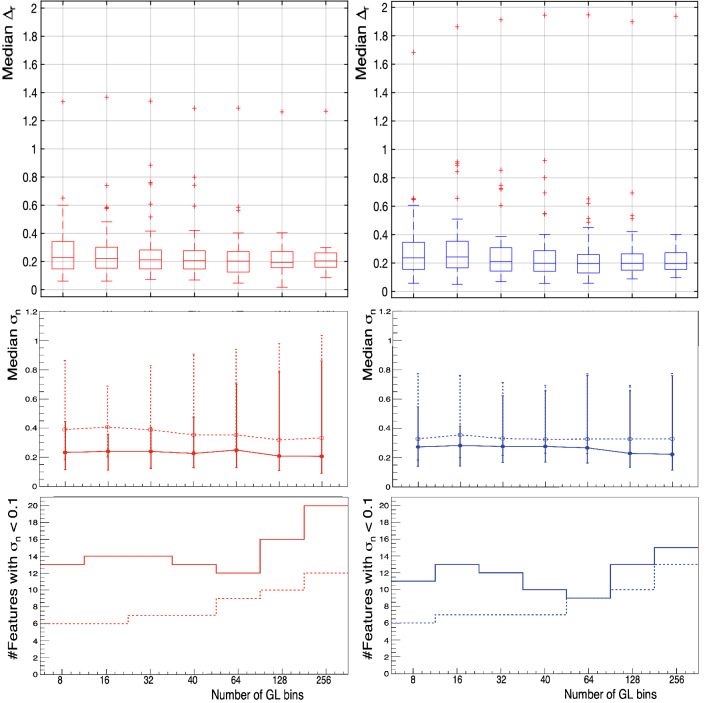
Bottom row: number of features with small or near-zero variance ($\sigma _n < 0.1$). Middle row: median of $\sigma _n$ (marker) and the first and third quartiles (lower and upper error bars, respectively) for the ensemble of 70 correlated features. Top row: boxplots of $\Delta _r$ for those features with $\sigma _n > 0.1$. All panels: tumour is denoted in red, left column, and muscle in blue, right column, for 2.0 mm (solid line, filled marker) and 5.0 mm (dashed line, hollow marker).

Figure 3 presents a summary of the results with the different GL quantisations. Starting from the bottom row, the number of features for which $\sigma _{n} < 0.1$ are shown for tumour (red, left column) and muscle (blue, right column) for 2.0 mm (solid, filled) and 5.0 mm (dashed, hollow). Those are the features considered to have a small standard deviation (or variation within patients), as it is smaller than $10\%$ of the value of the feature ($10\%$ being an arbitrary but reasonable value chosen for the purpose of this analysis). The middle row contains the median of $\sigma _n$ (marker) and the first and third quartiles (lower and upper error bars) for the ensemble of 70 features correlated with the number of GLs. The top row presents the boxplots of $\Delta _r$ for those features with $\sigma _n > 0.1$. There is consistently an outlier value far from the rest in the boxplots, which corresponds to the *GLCM* feature *ClusterShade*.

Results from Fig.3 indicate that the optimal quantisation should be chosen in the range (32–64) and that the result of the Freedman–Diaconis rule (40 bins) was found to indeed be a reasonable choice and was the one used in deriving the results described in the subsequent parts of this paper.

#### Correction of volume dependencies

The value of each radiomic feature was computed per patient and slice thickness (2.0 mm or 5.0 mm) with a total of 22 different variations of voxel sizes (1 original voxel sizes + 7 different resampled voxel sizes $\times$ 3 interpolation methods), maintaining the number of GLs fixed to 40 bins. With this ensemble of extracted values, the correlation between each radiomic feature and the number of voxels was analysed.

The Spearman’s rank correlation coefficient $r_S$ was calculated between radiomic feature values and number of voxels for each voxel size option. This showed that the most extreme values tested, namely the smallest (0.5, 0.5, 0.5) mm and the largest (1.75, 1.75, 1.75) mm and (2.0, 2.0, 2.0) mm, presented either an unnatural behaviour or provided incomplete information for certain features. This is illustrated in Supplementary Figure SF4, and shows some examples of features for which the values of $r_S$ considerably differs when using either the smallest voxel size (e.g. *GLCM* *Imc1* and *GLDM* *DependenceEntropy*) or the largest voxel size (e.g. *GLCM* *DifferenceEntropy* and *GLRLM* *RunEntropy*). This was found to be due to changes, sometimes subtle, in the values of the features that affect especially those cases with fewer number of voxels as presented in Supplementary Figures SF5-SF8. In those cases, where only information from few voxels was available, merging them into very large voxel sizes caused the number of entries in the GL matrices to be too few for feature calculations. Contrarily, using a very small voxel size increases the number of voxels used, which caused the GL matrices to become too sparse to provide reliable information. For these reasons, extreme values should be discarded when selecting the optimal voxel size, and they will not be taken into account in the remaining studies in this paper.

Features were deemed to be correlated with the number of voxels based on their Spearman’s rank correlation coefficient using a threshold of $r_S > 0.5$. Shape features were not taken into account as by definition they are the ones naturally containing information about the tumour volume. A total of 60 texture features were identified as correlated with the number of voxels, as shown in Supplementary Table ST5. In that table, the median value of $r_S$ across the distributions with different voxel sizes and interpolators is given for tumour and muscle, using 2.0 mm and 5.0 mm slice thickness images.

**Figure 4:**
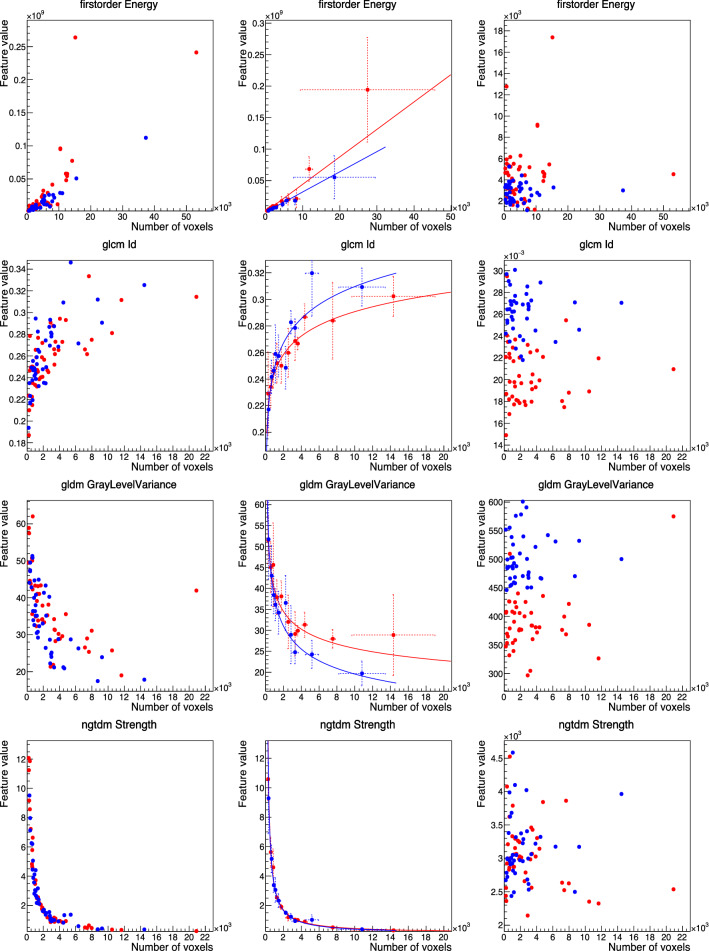
Examples of fits performed to four different features (one per row) that are correlated with the number of voxels, both for tumour (red) and muscle (blue), using images with 2.0 mm slice thickness and their original in-plane resolutions. In the left column, the feature values are shown before correction (one marker per patient), and on the right after correction. In the middle column, the result of the best fit performed on top of the intermediate graph is shown. Features are, from top to bottom: *first-order* *Energy* (best-fit function $[p1]\cdot x + [p0]$); *GLCM Id* (best-fit function $[p1]\cdot \log (x) + [p0]$); *GLDM GrayLevelVariance* (best-fit function $[p1]/\log (x) + [p0]$); *NGTDM Strength* (best-fit function $[p1]/x + [p0]$).

For those features found to be correlated, fits were performed testing the different functions listed in Materials and Methods. Some examples of the resulting best-fits can be found in Fig.4, where the feature values are shown before correction on the left column, the intermediate graph and result of the best fit is presented in the middle column, and the values of the features after correction (using the inverse function of the best fit) is shown in the right column.

**Figure 5:**
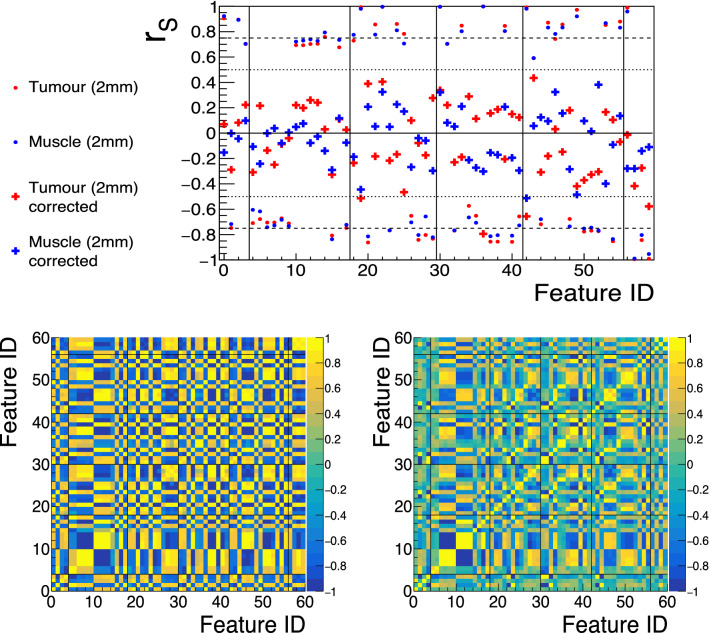
Illustration of the effect of correcting the dependencies of features with the number of voxels. Top: the Spearman’s rank correlation coefficient of the correlated features before (rounded markers) and after (cross markers) correcting, for both tumour (red) and muscle (blue) in images with 2.0 mm slice thickness and using their original voxel sizes. Bottom: the Spearman’s rank correlation coefficient showing pair-wise correlations between the features before (left) and after (right) the correction, for tumour and 2.0 mm slice thickness. Similar results are obtained when computing the correlation matrices using images with 5.0 mm slice thickness (as shown in Supplementary Figure SF9) and when computing them for muscle (as shown in Supplementary Figure SF10). Feature ID in this Figure follows the one in Supplementary Table ST5.

Corrections were implemented by using the inverted function corresponding to the best fit per feature, e.g. for a best-fit function $f(x) = [p1] \cdot \log (x) + [p0]$, the value of the feature was corrected by using $f'(x) = (f(x)-[p0])/\log (x)$. With the corrected features, the correlation with the number of voxels was expected to diminish, and this is confirmed in Fig. 5 (top), which shows $r_S$ before (hollow markers) and after (solid markers) the correction of the number of voxels dependencies. An important effect of correcting such dependencies is that the intrinsic dependencies between the features themselves, often used in a pre-processing step to remove redundant features in analyses, decreased as a consequence of reducing the dependency with the common factor (i.e. volume). This is also presented in Fig. 5 (bottom), that shows the pair-wise Spearman’s rank correlation coefficients before (left) and after (right) the number of voxels dependency correction.

### Robustness of radiomic features for different voxel sizes and slice thicknesses

The values of the texture radiomic features, computed independently in the set of images with 2.0 mm and 5.0 mm with the 22 different voxel size settings described previously, were compared to assess their robustness. The metric $\Delta _r$ as in Eq. (4) was used for this purpose.

**Figure 6:**
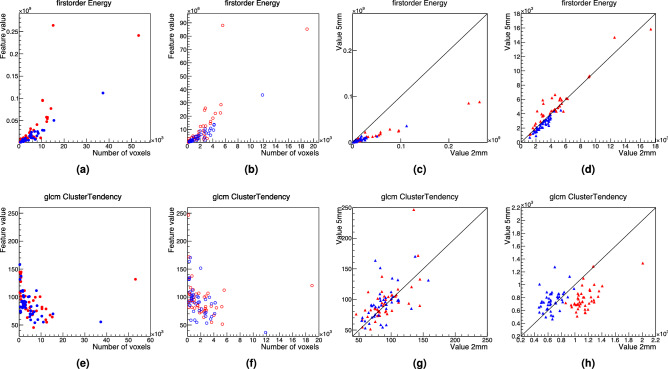
Example of the comparison of the values with different slice thicknesses of two features: *first-order Energy* (top row) and *GLCM ClusterTendency* (bottom row). The values of the features in images with 2.0 mm slice thickness are presented in (**a**) (*Energy*) and (**e**) (*ClusterTendency*) and with 5.0 mm slice thickness in (**b**) and (**f**) for tumour (red) and muscle (blue). The values calculated with 2.0 mm and 5.0 mm are compared in a case by case basis in (**c**) and (**g**) before correcting for the dependency with the number of voxels, and in (**d**) and (**h**) after correcting for it, showing that in one case (*Energy*) the agreement is better after correcting whilst in the other (*ClusterTendency*) is not.

The dependency of features with the number of voxels was first considered and a correction for this dependency examined. A correction was found to be necessary for some features, for example the *first-order Energy* feature (which is an additive measurement of the magnitude of voxel values). For others, e.g. *GLCM ClusterTendency* (which is a measurement of heterogeneity), the difference of its value per patient between slice thicknesses was insignificant. To illustrate this, Fig.6 shows details for these two features as examples. Figure 6a–d relate to the feature *first-order Energy*, with its value as a function of the number of voxels presented in Fig. 6a (Fig. 6b) for 2.0 mm (5.0 mm) slice thickness for tumour (red) and muscle (blue). Clearly, the range of the feature value (Y axis) is very different for Fig. 6a,b; both values are compared patient by patient in Fig. 6c, showing the striking difference in value at 2.0 mm and 5.0 mm slices. However, after correcting for the dependency on the number of voxels, both values become very similar as can be easily appreciated in Fig. 6d. Similar plots are presented for the feature *GLCM ClusterTendency* in Fig. 6e–h. In this case, however, there is no difference in the feature values (Y axis) between 2.0 mm (Fig. 6e) and 5.0 mm (Fig. 6f), meaning that the value of this feature is (with some exceptions) very similar between both slice thicknesses, as clearly shown in Fig. 6g. Since the number of voxels is however different for 2.0 mm (Fig. 6e) and 5.0 mm (Fig. 6f), an attempt to correct for the dependency in the number of voxels was found to have a detrimental impact.

The value of the metric $\Delta _r$ ($\Delta _r > 0.5$) was used to decide the features to correct for their dependency with the number of voxels before comparing their values in both slice thicknesses. A total of 14 features were found to need such a correction as presented in Supplementary Tables ST8-ST9 (in the original, no re-sampled, voxel sizes); these were: (*first-order*) *Energy*, (*GLCM*) *Imc1*, (*GLDM)* *DependenceNonUniformity*, *GrayLevelNonUniformity*, (*GLRLM*) *GrayLevelNonUniformity*, *RunLengthNonUniformity*, (*GLSZM*) *GrayLevelNonUniformity*, *LargeAreaEmphasis*, *LargeAreaHighGrayLevelEmphasis*, *ZoneVariance*, (*NGTDM*) *Busyness*, *Coarseness*, *Contrast*, *Strength*. After correcting their dependencies with the number of voxels, 11 of them (all except *LargeAreaEmphasis*, *LargeAreaHighGrayLevelEmphasis* and *ZoneVariance*) had values Δr<0.5.

**Figure 7:**
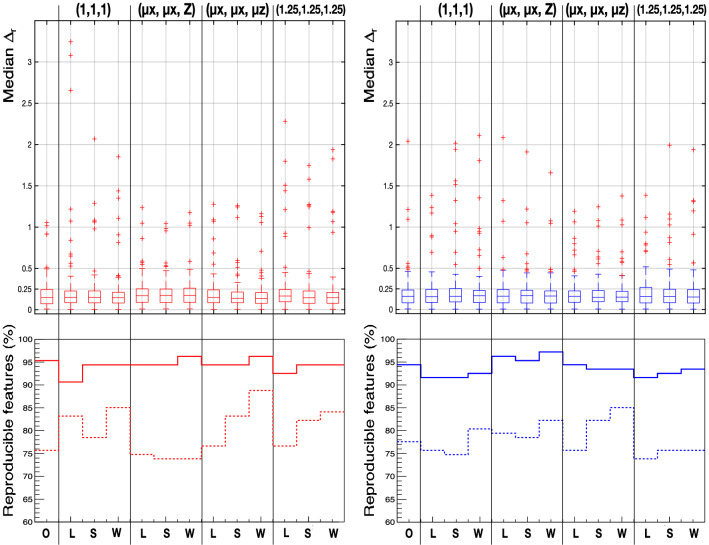
Top row: boxplots conveying the information about the median (across all patients) values of $\Delta _r$ for all features, calculated for tumour (red, left) and muscle (blue, right) for the different combinations of voxel sizes (as shown on the top) and interpolation methods (*O* original, *L* linear; *S* B-spline, *W* Welch). Bottom row: percentage of the whole ensemble of 107 features that can be deemed robust across changes in slice thickness ($\Delta _r < 0.5$, solid lines) and highly robust ($\Delta _r < 0.25$, dashed lines) for each re-sampling option. Some features are corrected for their dependency on the number of voxels before the comparison between slice thicknesses is made. More details can be found in the text.

A summary of the values of $\Delta _r$ after the corrections explained above is given in Fig.7, where the boxplots convey the information for all features of their median values of $\Delta _r$ within the cohort of patients for tumour (red, left) and muscle (blue, right) for the different combinations of voxel sizes and interpolation methods. In addition, the histograms in the bottom row of Fig.7 show the number of features that can be deemed robust ($\Delta _r < 0.5$, solid lines) and highly robust ($\Delta _r < 0.25$, dashed lines) for each re-sampling option. Most features (75%–90%) were found to be highly robust, i.e. with a difference between the values of less than $25\%$.

Although there was no significant difference found between the median values of the boxplots, there were differences in the number of robust, and especially highly robust features, between the different voxel sizes, and even between the different interpolation methods for a given voxel size. In general, higher robustness was obtained when using a voxel size with a z-dimension intermediate to (optimally, the mean) those of the two slice thicknesses. For each given fixed voxel size, it was also found a higher level of robustness was achieved using the Welch windowed sinc interpolation method. Therefore, from Fig.7 it could be inferred that the voxel size ($\mu _x$, $\mu _y$, $\mu _z$) and the Welch windowed sinc interpolation method was optimal to reduce the discrepancy between the values of the features obtained with images of 2.0 mm and 5.0 mm slice thickness.

### Normalisation using healthy tissue

The robustness of texture radiomic features was investigated using healthy tissue to normalise values following Eq. (6), and by constructing and comparing the metric $\Delta _{\rho }$ from Eq. (7).

**Figure 8:**
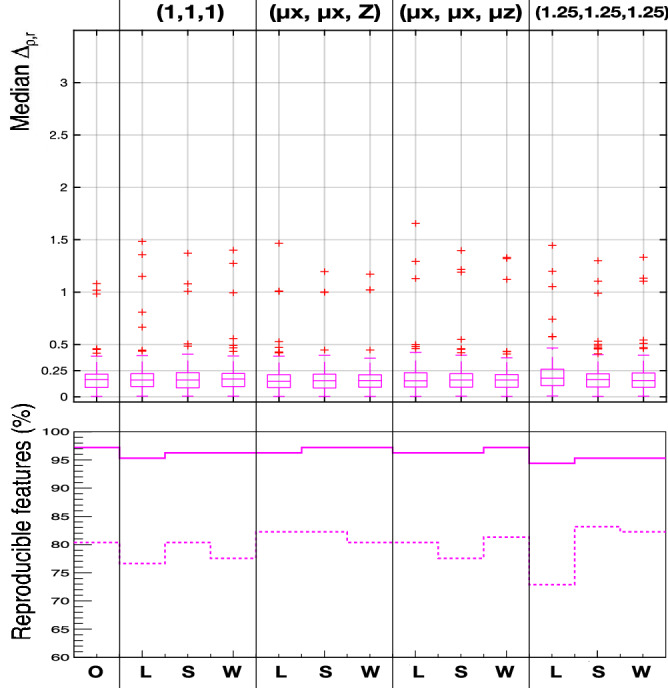
Top row: median values of $\Delta _{\rho ,r}$ ($\Delta _\rho$ and $\Delta _r$ for texture and shape features, respectively), comparing the difference between the ratios of the value of each feature in tumour over muscle in 2.0 mm and 5.0 mm, for the different combinations of voxel sizes (as shown on the top) and interpolation methods (*O* original; *L* linear; *S* B-spline, *W* Welch). Bottom row: percentage of the whole ensemble of 107 features that can be deemed robust ($\Delta _{\rho ,r} < 0.5$, solid lines) and highly robust ($\Delta _{\rho ,r} < 0.25$, dashed lines) for each re-sampling option.

The result of this comparison is presented in Fig.8, which shows a boxplot of the median value within the cohort of patients of $\Delta _{\rho }$, for all the ensemble of 107 radiomic features for each voxel size and interpolation method combination. The previously used metric $\Delta _{r}$ was maintained in Fig.8 for the shape features, whilst the others are compared in terms of ratios with $\Delta _{\rho }$. In addition, the percentage of features that are robust ($\Delta _{\rho ,r} < 0.5$) and highly robust ($\Delta _{\rho ,r} < 0.25$) are also presented. The median values for each feature can be found in Supplementary Tables ST10-ST11.

This test shows that the percentage of robust features that can be achieved was similar to that in Fig.7, and it was more stable across the different voxel sizes and interpolation methods used. However, the number of highly robust features was slightly worse than the number that could be achieved with the optimal normalisation choice shown in Fig.7 (i.e. with ($\mu _x$, $\mu _y$, $\mu _z$) and the Welch windowed sinc interpolation, and the correction of the dependencies with the number of voxels).

## Discussion

In this paper, important considerations for the computation of radiomic features for a dataset of heterogeneous voxel size and slice thickness CT images were described. The effect of the intrinsic dependencies of radiomic features was analysed, ways to correct for these dependencies were developed and optimal choices were identified.

First of all, the impact of the GL quantisation on the features was studied (summarised in Fig.3). On the one hand, although there is not a remarkable variation in the values of $\Delta _r$ (as described in Eq. 4), its median and IQR are larger for the smaller number of GLs tested (8, 16 bins). On the other hand, the number of features with small ($\sigma _n < 0.1$) variance increases for the largest values tested (i.e. 128, 256 bins), as does the IQR range of variance $\sigma _n$. This shows that the number of GL bins affects the values computed for some features, such that they might “collapse” to a single value for all patients if the number of bins is not large enough to account for differences in neighbouring pixels (i.e. with the smaller values of 8 and 16 bins) or too large to produce meaningful comparisons of the pixel intensities in the GL matrices (i.e. with the larger values of 128 and 256 bins). It was therefore found that the optimal range of GLs was (32–64), and that the Freedman–Diaconis rule could be used to find a reasonable choice for this quantisation. These conclusions hold for both tissue types, tumour and muscle. It is worth mentioning that the default bin width of PyRadiomics has a value of 25, which for the ranges of HU of the tumour and muscle ROIs in this cohort would translate into $\sim 6$ bins, clearly not optimal for the computation of features in light of these results.

Secondly, the correlation of radiomic features with the number of voxels was analysed and corrected. It was important to correct this dependency when making comparisons across different sized regions. Pair-wise correlations amongst the features were found to decrease in strength after this correction, since a common factor (their dependency on the volume or number of voxels) had been eliminated. Such correction is therefore necessary to understand the actual intrinsic dependencies between the features, which is often employed in pre-processing steps to remove redundant features from the analysis.

It is important to note that shape-based features were purposely not corrected in this paper, as those features must convey by definition information about the shape and therefore the volume. It was not the goal of the studies presented, nor the recommended approach, to completely remove any information about the volume of the ROIs, which might be indeed relevant for the analyses performed, but to instead preserve it only in the features where it is inherent, such as in shape-related features like lengths and diameters.

Lastly, the robustness of the features computed with different reconstructed slice thicknesses was investigated both in liver tumour and healthy muscle tissue. Interestingly, it was found that most features were highly robust, but that the number of robust features did depend on the voxel size and interpolation method used, being the optimal choice using a voxel size with a z-dimension intermediate to (optimally, the mean) those of the two slice thicknesses, with the Welch windowed sinc interpolation method. Some features were found to never be robust between both slice thickness values.

A further study was performed to investigate results using normalisation with healthy (muscle) tissue reported in prior literature^31–34^. In the study presented in this paper, the texture features of the liver tumour were normalised using the corresponding texture values from healthy (muscle) tissue. It was found that such a normalisation could be an alternative approach to achieve robust (across slice thickness) texture feature results, stable across different voxel sizes and interpolation methods. Robustness results in this case were adequate but slightly worse than those achieved using the feature correction method described above.

Existing literature work has investigated the impact of intrinsic dependencies and acquisition parameters on radiomic feature extraction. The majority of these studies focus on CT, PET and MRI in oncological applications. Shafiq-ul-Hassan et al.^14^ addressed the impact of slice thickness and pixel spacing, along with other acquisition and reconstruction parameters, on a subset of radiomic features extracted from phantom images acquired using different CT scanners. The same research group also considered feature dependency on GL quantisation in a later study^35^, validating on lung tumour CT images the voxel size and GL normalisations previously found in phantom studies. Other studies have investigated feature robustness using image perturbations, like Zwanenburg et al.^36^ by adding noise, as well as performing translations or rotations. Moreover, the ROIs were perturbed by volume erosion/dilation and supervoxel-based contour randomisation. Test-retest and perturbation robustness were evaluated on two CT datasets: (1) non-small-cell lung cancer (NSCLC), and (2) head-and-neck squamous cell carcinoma (HNSCC). Leijenaar et al.^37^ focused on the implications of the quantisation step in PET-based radiomic features. In particular, the standardised uptake value (SUV) quantisation showed a crucial effect on the resulting textural features and the corresponding interpretation. This experimental evidence pointed out the importance of standardised procedures for tumour texture analysis.

Several other studies have analysed the robustness of MRI-based radiomic features. These span from phantom studies where various aspects were analysed (signal-to-noise ratio, ROI delineation, voxel sizes and normalisation methods)^38^ to oncology reports on reproducibility and repeatability. Reproducibility was assessed by either using different normalisation methods^31^ or estimating the inter-operator variability in various contexts, namely when manual segmentation^39^, automatic perturbed delineations (with the goal of simulating the inter-observer variability)^40^, or semi-automatic and interactive segmentation solutions were applied^41^. Repeatability improvements were also studied by applying several pre-processing and extraction configurations (such as image normalisation schemes, image pre-filtering and bin widths) in multiparametric MRI scans of small prostate tumours^32^, however without definitive recommendations.

All these radiomic robustness studies leveraged classical statistical methods, such as the ICC, Spearman’s correlation coefficient, coefficient of variation and Bland-Altman analysis. The inter-observer variability was typically evaluated by the Dice Similarity Coefficient (DSC). In this paper, a novel methodological approach was introduced, based on testing invertible functions to fit the underlying dependency of each feature with respect to the number of voxels, to analytically correct for the observed dependencies in an automatic and software-agnostic way. In addition to analyse the impact of the GL quantisation and voxel size used in the computation of the features like existing approaches, this work aimed to justify optimal choices for such parameters in the context of images with different slice thicknesses, and to provide guidelines. Finally, the question of how many features are robust with respect to a change in slice thickness is addressed, which to best of our knowledge has not been studied so far. All these studies were performed with a consistent comparison between tumour and healthy (i.e. muscle) tissue, a practice not common in previous literature studies.

The main limitation of this analysis was the small size of the cohort used (43 cases). This was sufficient to perform suitable fits, but they would have been improved with a larger cohort size: it would have allowed for further optimisation of the number of bins in the intermediate graph to fit and would have provided additional statistics, especially in regions in which the current cohort did not have available cases. It is important to note, however, that the sample size used in this analysis is representative of patient sample sizes generally reported in the literature.

Future work should analyse the effect of other CT acquisition parameters on the robustness and reproducibility of radiomic features, and their impact in radiomics-based models to characterise liver cancer and, by extension, other cancer types.

In conclusion, the following guidelines can be proposed in light of the results. They apply to a CT-based radiomic analysis in a cohort heterogeneous in terms of slice thickness and in-plane resolution, and they have been tested on HCC tumours and muscle tissue (work to generalise to other cancer types should follow):

- Too small or too large GL quantisations (such as 8 or 256 bins) should be avoided, as they might impact the values of the texture features by significantly reducing the information available in their computation.
- The result of the Freedman–Diaconis statistics rule on a given cohort gives an indication of a reasonable value of the number of GLs to be chosen for the computation of radiomic features.
- It is important to correct texture features for their dependency on the number of voxels (or volume) before they can be compared within a heterogeneous cohort. For example the commonly used *first-order* feature *Energy* has a strong volume dependence that yields a very different value for the same tumour in images reconstructed with two different slice thicknesses. Volume dependencies should also be corrected before correlations between the features themselves can be investigated.
- For interpolation, a voxel size should be selected with a size approximately the average of that over the range of values of the cohort. The Welch sinc interpolation method should be used in preference to linear or B-spline methods.
- Alternatively, a normalisation approach can be performed by using the ratios of the feature values calculated in the tumour to the respective values from similarly sized muscle regions.

## Supplementary information

Supplementary Informations.

## Data Availability

The datasets generated and analyzed during this study are available from the corresponding author on reasonable request.
