# Supplementary material for: Robustness of radiomic features in CT images with different slice thickness, comparing liver tumour and muscle
Source: Sci Rep. 2021 Apr 15;11:8262. doi: 10.1038/s41598-021-87598-w (PMC8050292; doi:10.1038/s41598-021-87598-w)
Supplement: Supplementary file 1 — Supplementary Informations. [file 41598_2021_87598_MOESM1_ESM.pdf]

Robustness of radiomic features in CT images with different slice thickness, comparing liver tumour and muscle

February 5, 2021

Lorena Escudero Sanchez, Leonardo Rundo, Andrew B. Gill, Matthew Hoare, Eva Mendes Serrao, Evis Sala

Supplementary Material

List of Figures

SF1 Radiomic features (Y) for different number of grey levels used in the calculation (X) in CT images with 2.0mm slice thickness, resampled to voxel size (1,1,1) mm, for liver tumour (red) and muscle (blue). Each dot (marker) represents one patient. 7
SF2 Radiomic features (Y) for different number of grey levels used in the calculation (X) in CT images with 2.0mm slice thickness, resampled to voxel size (1,1,1) mm, for liver tumour (red) and muscle (blue). Each dot (marker) represents one patient. 8
SF3 Radiomic features (Y) for different number of grey levels used in the calculation (X) in CT images with 2.0mm slice thickness, resampled to voxel size (1,1,1) mm, for liver tumour (red) and muscle (blue). Each dot (marker) represents one patient. 9
SF4 Examples of features that present a pathological behaviour for extreme values of the resampling, either for the smallest one tested, (0.5,0.5,0.5)mm, in the top row, or the largest ones, (1.75,1.75,1.75)mm and (2,2,2)mm, in the bottom row. This behaviour is observed through a significant change of the r_S (Y axis) extracted from the feature values as a function of the number of voxels (see Supplementary Figures SF5-SF8). Each TestID represents a different voxel size, with 0 being the original (i.e. no resampling) and following triadas of linear-spline-welch interpolation methods. . . 10
SF5 Values of the feature GLCM IMC1 (each marker represents a patient) for tumour (red) and muscle (blue) in images with 2mm (solid markers, top two rows) and 5mm (hollow markers, bottom two rows) slice thickness. A comparison is presented for the values computed with a voxel size of (0.5,0.5,0.5)mm on the left column, vs the original voxel sizes on the middle column, and (2.0,2.0,2.0)mm on the right column. 11
SF6 Values of the feature GLDM DependenceEntropy (each marker represents a patient) for tumour (red) and muscle (blue) in images with 2mm (solid markers, top two rows) and 5mm (hollow markers, bottom two rows) slice thickness. A comparison is presented for the values computed with a voxel size of (0.5,0.5,0.5)mm on the left column, vs the original voxel sizes on the middle column, and (2.0,2.0,2.0)mm on the right column. 12
SF7 Values of the feature GLCM DifferenceEntropy (each marker represents a patient) for tumour (red) and muscle (blue) in images with 2mm (solid markers, top two rows) and 5mm (hollow markers, bottom two rows) slice thickness. A comparison is presented for the values computed with a voxel size of (0.5,0.5,0.5)mm on the left column, vs the original voxel sizes on the middle column, and (2.0,2.0,2.0)mm on the right column. 13

SF8 Values of the feature GLRLM RunEntropy (each marker represents a patient) for tumour (red) and muscle (blue) in images with 2mm (solid markers, top two rows) and 5mm (hollow markers, bottom two rows) slice thickness. A comparison is presented for the values computed with a voxel size of (0.5,0.5,0.5)mm on the left column, vs the original voxel sizes on the middle column, and (2.0,2.0,2.0)mm on the right column. 14
SF9 Matrices of Spearman's rank correlation coefficient showing pairwise correlations between the features before (left column) and after (right column) correcting the dependencies of features with the number of voxels for the **tumour** VOIs calculated in CT images with reconstructed slice thickness of 2mm (top row) and 5mm (bottom row) and the original voxel size (i.e. no resampling). Feature ID in this Figure follows the one in Supplementary Table ST3. 15
SF10 Matrices of Spearmans rank correlation coefficient showing pairwise correlations between the features before (left column) and after (right column) correcting the dependencies of features with the number of voxels for the **muscle** VOIs calculated in CT images with reconstructed slice thickness of 2mm (top row) and 5mm (bottom row)and the original voxel size (i.e. no resampling). Feature ID in this Figure follows the one in Supplementary Table ST3. 16

List of Tables

ST1 CT acquisition parameters for the images used in this paper. . . 6

ST2 Patient demographics of the studies analysed in this paper. . . . 6

ST3 Values of ICC(3,1) for muscle and tumour, in 2mm and 5mm slices, independently. Those features with ICC(3,1)< 0.9 are considered correlated, except the ones shown in blue, for which only one value appears just below 0.9 in the four cases, and therefore are treated as fluctuations. 17

ST4 Values of ICC(3,1) for muscle and tumour, in 2mm and 5mm slices, independently. Those features with ICC(3,1)< 0.9 are considered correlated, except the ones shown in blue, for which only one value appears just below 0.9 in the four cases, and therefore are treated as fluctuations. 18

ST5 Median values of Spearman's rank correlation coefficient (r_S), across different non-extreme voxel size resampling and interpolation method, for features found correlated ($r_S > 0.5$) with the number of voxels. High correlations are shown in red ($r_S > 0.75$) and medium in blue ($0.75 > r_S > 0.5$) 19

ST6 Median values of the relative difference between the values of the features computed in images with 2mm and 5mm slice thickness (Δ_r), across different non-extreme voxel size resampling and interpolation method, before correcting for the dependence with the number of voxels. Values $\Delta_r \geq 0.5$ are shown in red. 20

ST7 Median values of the relative difference between the values of the features computed in images with 2mm and 5mm slice thickness (Δ_r), across different non-extreme voxel size resampling and interpolation method, before correcting for the dependence with the number of voxels. Values $\Delta_r \geq 0.5$ are shown in red. 21

ST8 Median values of the relative difference between the values of the features computed in images with 2mm and 5mm slice thickness (Δ_r), across different non-extreme voxel size resampling and interpolation method, after correcting certain features for the dependence with the number of voxels.Values $\Delta_r \geq 0.5$ are shown in red. 22

ST9 Median values of the relative difference between the values of the features computed in images with 2mm and 5mm slice thickness ($\Delta_r o$), across different non-extreme voxel size resampling and interpolation method, after correcting certain features for the dependence with the number of voxels. Values $\Delta_r \geq 0.5$ are shown in red. 23

ST10Median values of the relative difference between the values of the feature ratios (tumour over muscle) computed in images with 2mm and 5mm slice thickness ($\Delta_{\rho,r}$), across different non-extreme voxel size resampling and interpolation method. Values $\Delta_{\rho,r} \geq 0.5$ are shown in red. 24

ST11 Median values of the relative difference between the values of the feature ratios (tumour over muscle) computed in images with 2mm and 5mm slice thickness ($\Delta_{\rho,r}$), across different non-extreme voxel size resampling and interpolation method. Values $\Delta_{\rho,r} \geq$ 0.5 are shown in red. 25

	**2mm series**	**5mm series**
**Vendor**	SIEMENS	
**Model**	Sensation 64 (19), Sensation 16 (5)<br>SOMATOM Definition Flash (19)	
**Reconstruction kernel**	B20f	B20f (19), B30f (24)
**Reconstruction diameter (mm)**	290 - 473<br>(average 376.16)	290 - 473<br>(average 376.23)
**KVP**	120	
**Exposure**	46 - 340<br>(average 150.19)	46 - 342<br>(average 149.39)
**Matrix size (pixels)**	512x512	
**Slice thickness (mm)**	2.0	5.0
**Pixel width (mm)**	0.566 - 0.924<br>(average 0.735)	

Table ST1: CT acquisition parameters for the images used in this paper.

	**Range**
**Patient age at transplant (years)**	41.88 - 67.60<br>(average 55.52)
**Patient gender**	Male (35), Female (8)
**Previous treatment**	1x Trans-arterial embolisation,<br>rest none
**Primary liver disease (prior to HCC)**	Hepatitis C cirrhosis (26),<br>Non-alcoholic fatty liver disease (6),<br>Alcoholic liver disease (4),<br>Hepatitis B cirrhosis (4),<br>Autoimmune hepatitis (1),<br>Cryptogenic cirrhosis (1),<br>Primary biliary cirrhosis (1)

Table ST2: Patient demographics of the studies analysed in this paper.

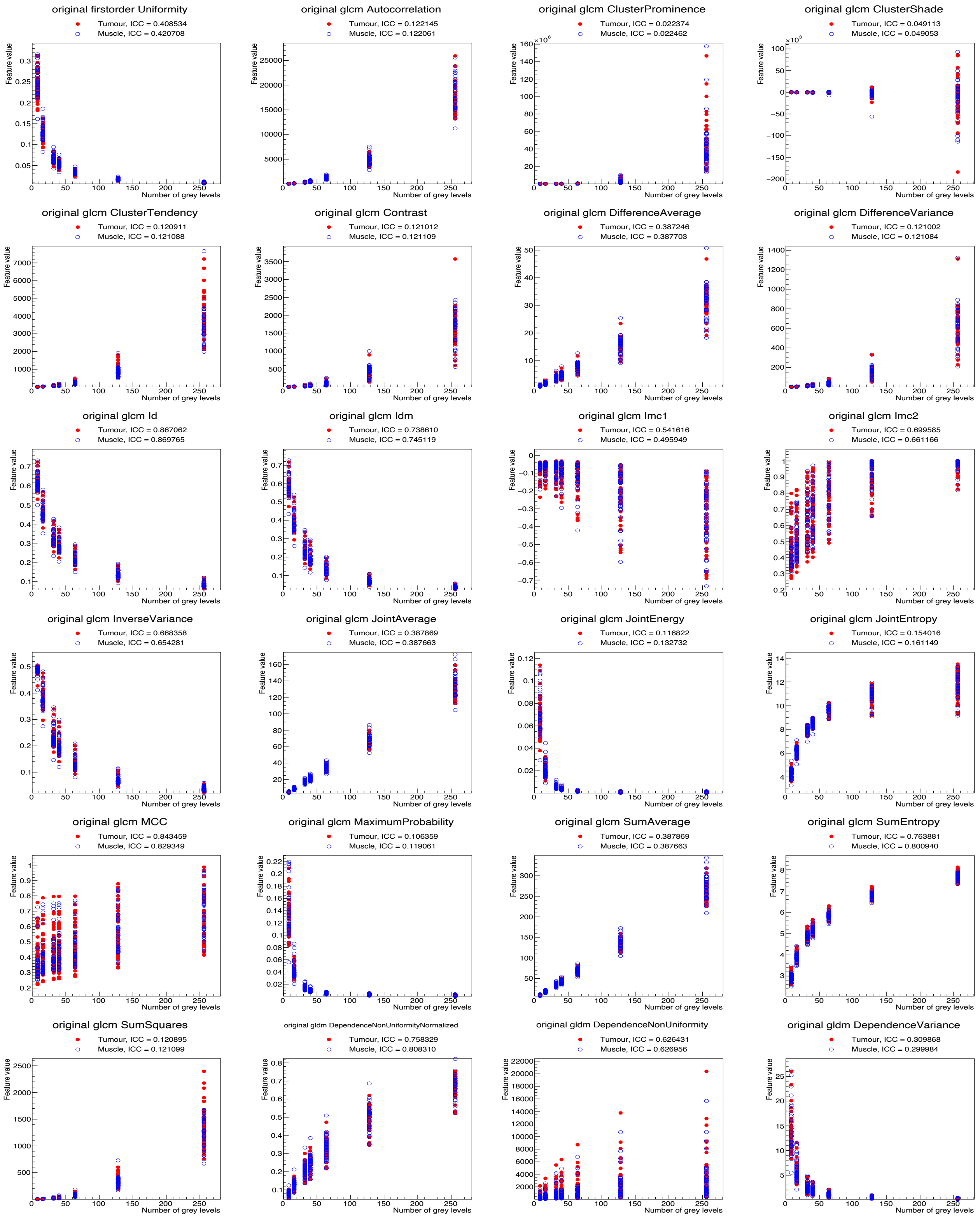

Figure SF1: Radiomic features (Y) for different number of grey levels used in the calculation (X) in CT images with 2.0mm slice thickness, resampled to voxel size (1,1,1) mm, for liver tumour (red) and muscle (blue). Each dot (marker) represents one patient.

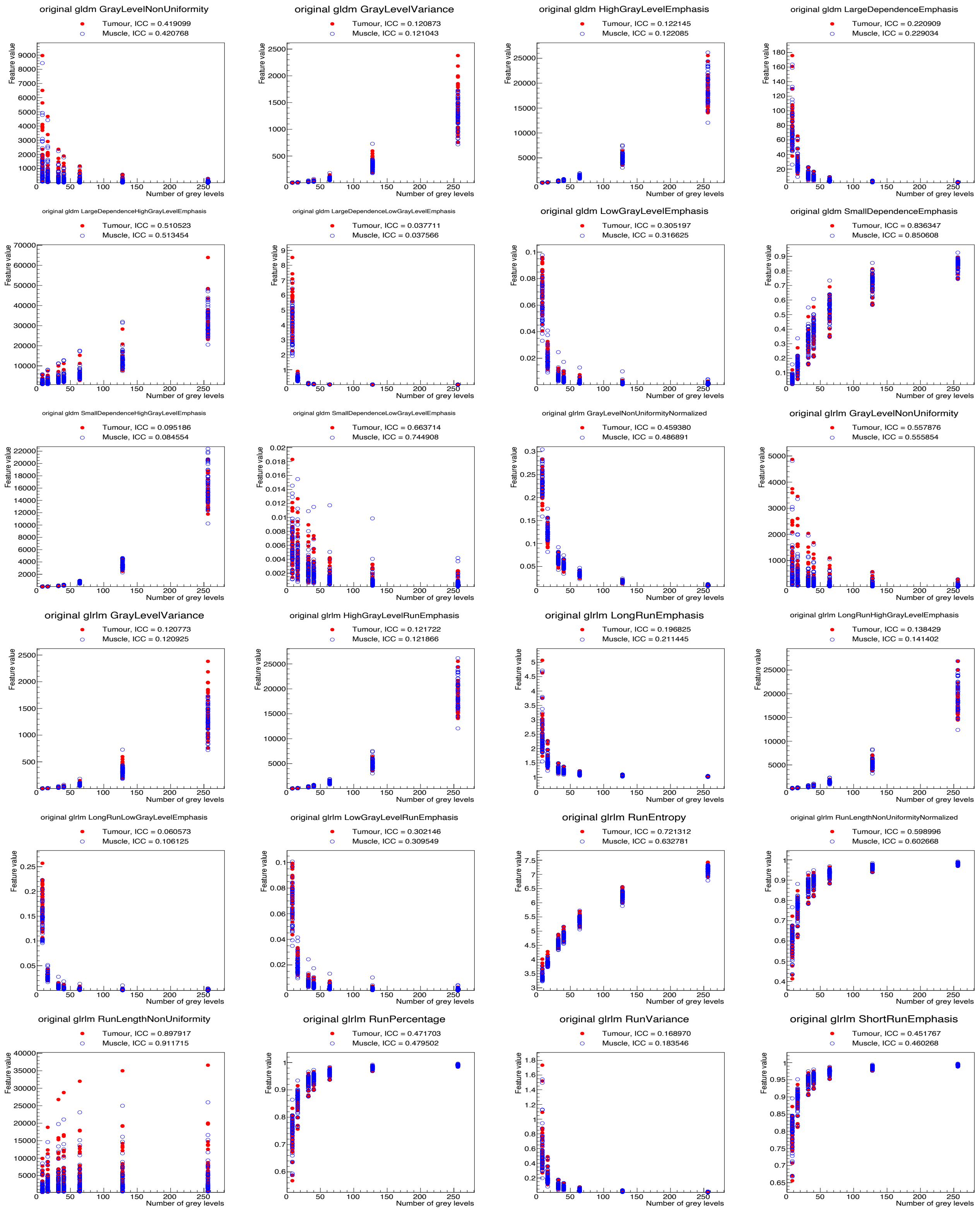

Figure SF2: Radiomic features (Y) for different number of grey levels used in the calculation (X) in CT images with 2.0mm slice thickness, resampled to voxel size (1,1,1) mm, for liver tumour (red) and muscle (blue). Each dot (marker) represents one patient.

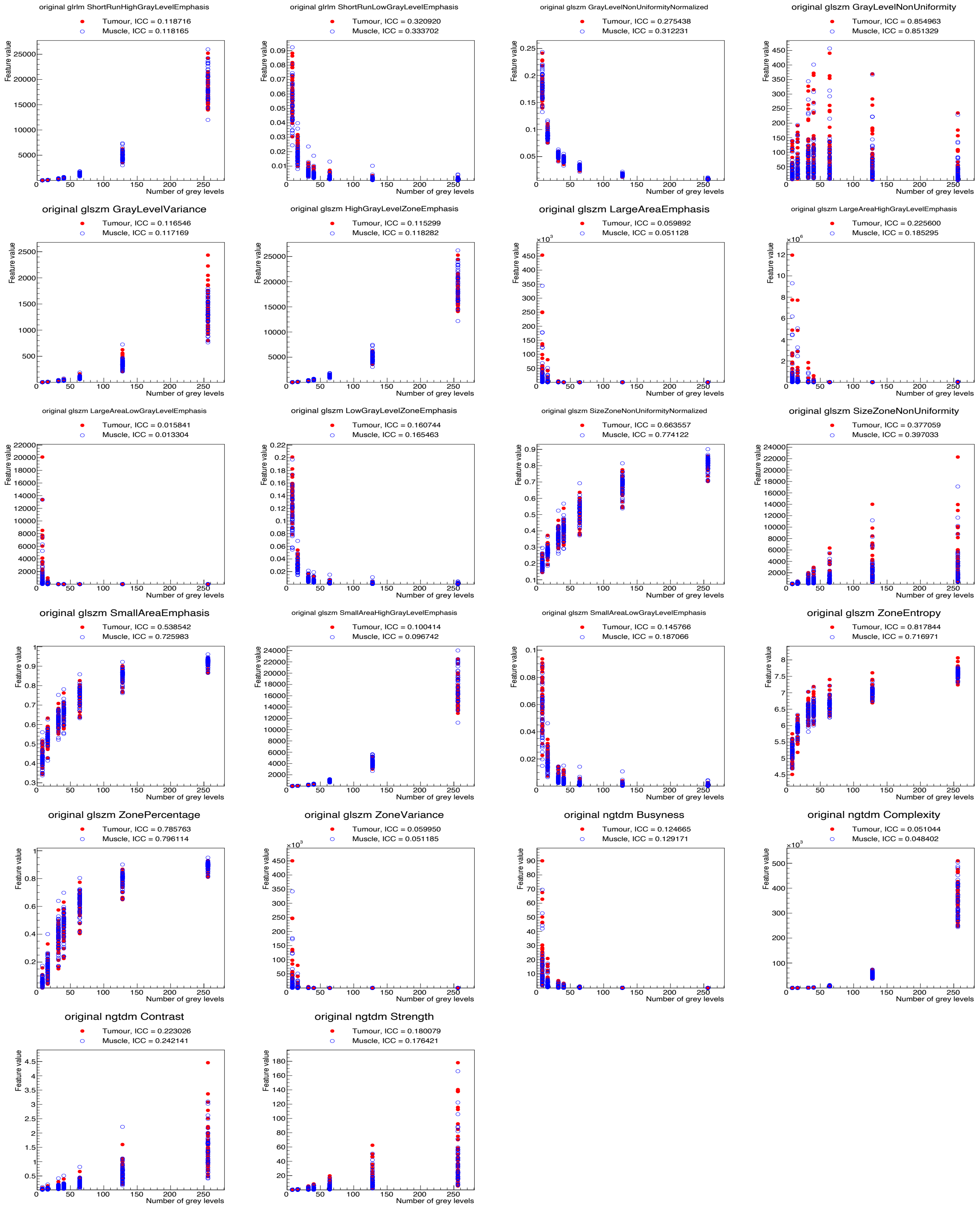

Figure SF3: Radiomic features (Y) for different number of grey levels used in the calculation (X) in CT images with 2.0mm slice thickness, resampled to voxel size (1,1,1) mm, for liver tumour (red) and muscle (blue). Each dot (marker) represents one patient.

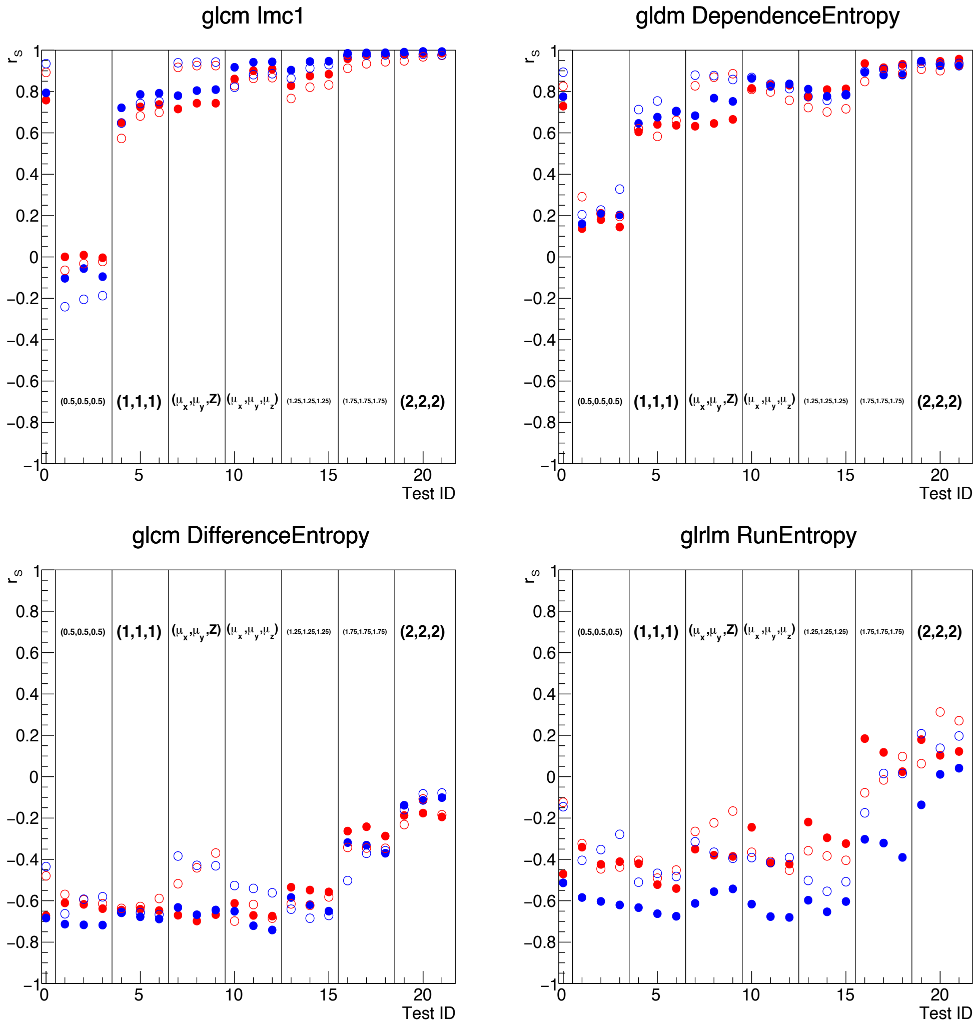

Figure SF4: Examples of features that present a pathological behaviour for extreme values of the resampling, either for the smallest one tested, (0.5,0.5,0.5)mm, in the top row, or the largest ones, (1.75,1.75,1.75)mm and (2,2,2)mm, in the bottom row. This behaviour is observed through a significant change of the r_S (Y axis) extracted from the feature values as a function of the number of voxels (see Supplementary Figures SF5-SF8). Each TestID represents a different voxel size, with 0 being the original (i.e. no resampling) and following triadas of linear-spline-welch interpolation methods.

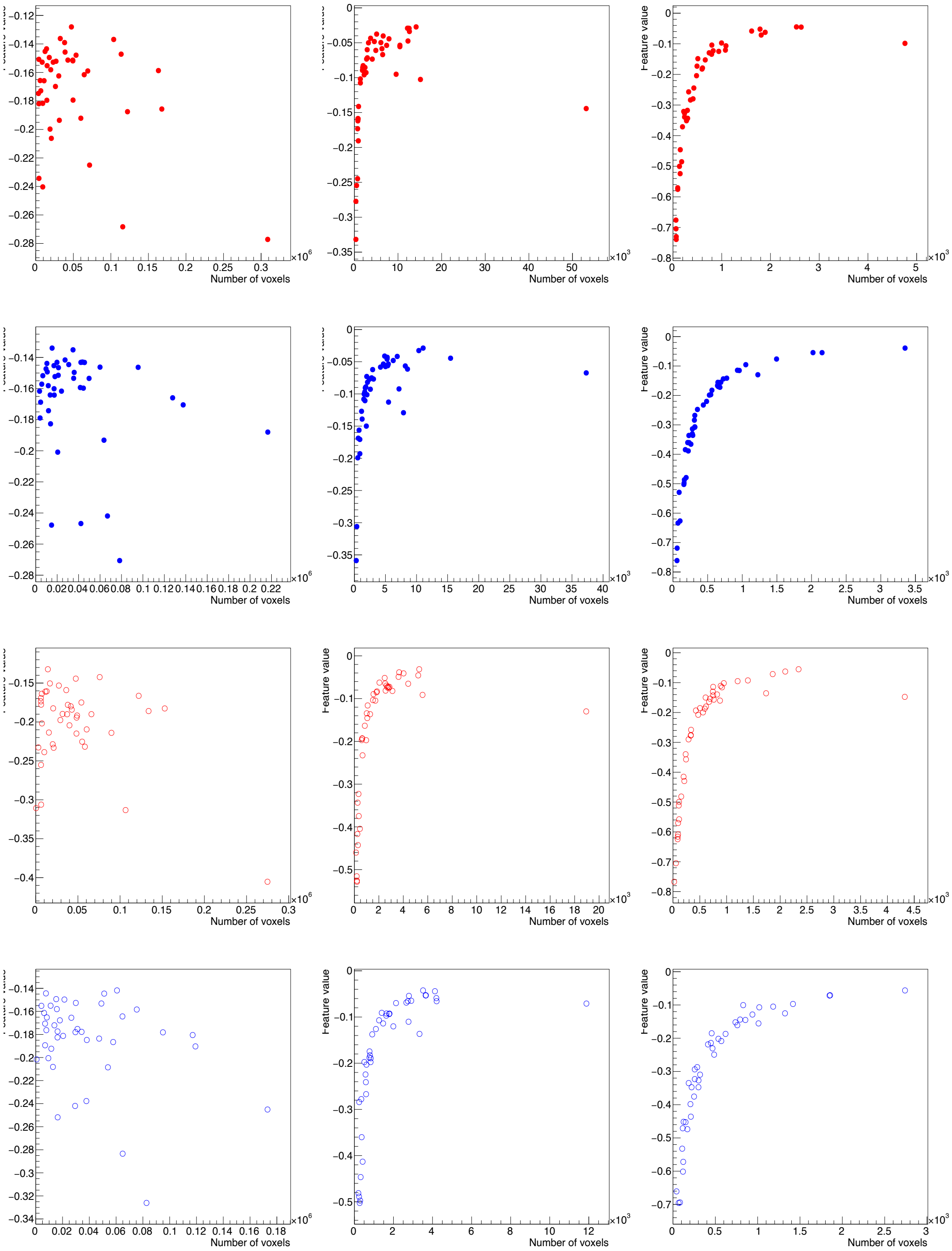

Figure SF5: Values of the feature GLCM IMC1 (each marker represents a patient) for tumour (red) and muscle (blue) in images with 2mm (solid markers, top two rows) and 5mm (hollow markers, bottom two rows) slice thickness. A comparison is presented for the values computed with a voxel size of (0.5,0.5,0.5)mm on the left column, vs the original voxel sizes on the middle column, and (2.0,2.0,2.0)mm on the right column.

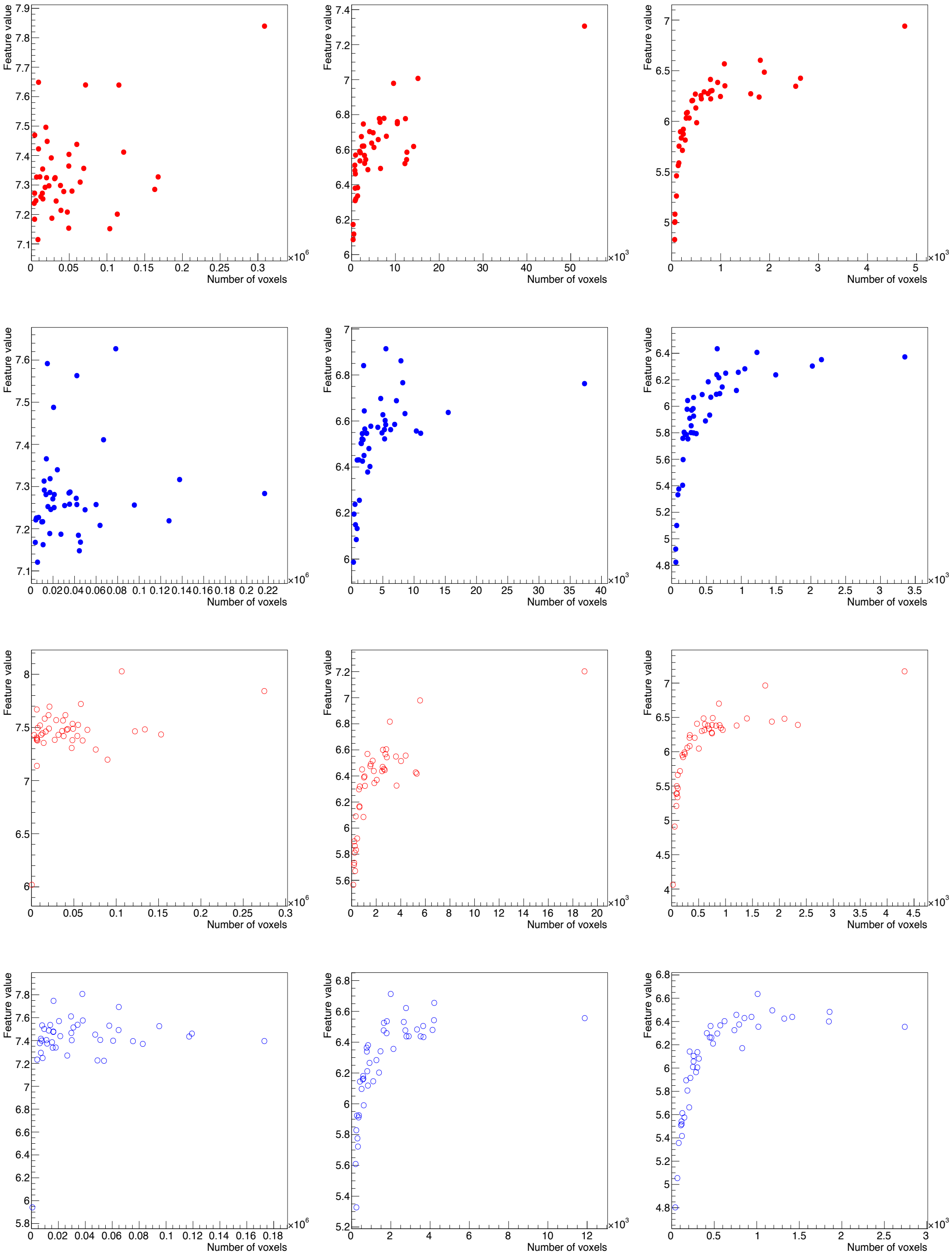

Figure SF6: Values of the feature GLDM DependenceEntropy (each marker represents a patient) for tumour (red) and muscle (blue) in images with 2mm (solid markers, top two rows) and 5mm (hollow markers, bottom two rows) slice thickness. A comparison is presented for the values computed with a voxel size of (0.5,0.5,0.5)mm on the left column, vs the original voxel sizes on the middle column, and (2.0,2.0,2.0)mm on the right column.

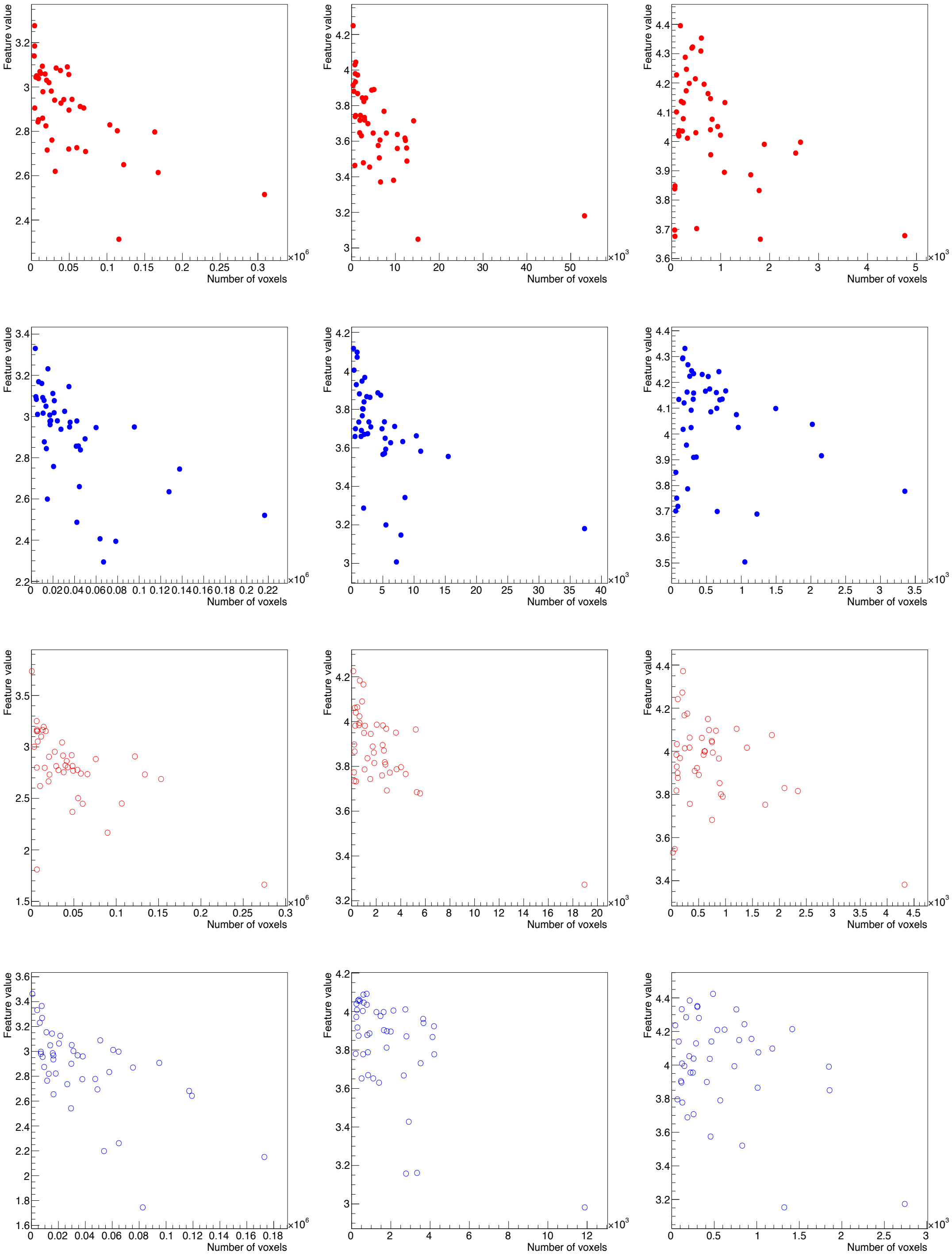

Figure SF7: Values of the feature GLCM DifferenceEntropy (each marker represents a patient) for tumour (red) and muscle (blue) in images with 2mm (solid markers, top two rows) and 5mm (hollow markers, bottom two rows) slice thickness. A comparison is presented for the values computed with a voxel size of (0.5,0.5,0.5)mm on the left column, vs the original voxel sizes on the middle column, and (2.0,2.0,2.0)mm on the right column.

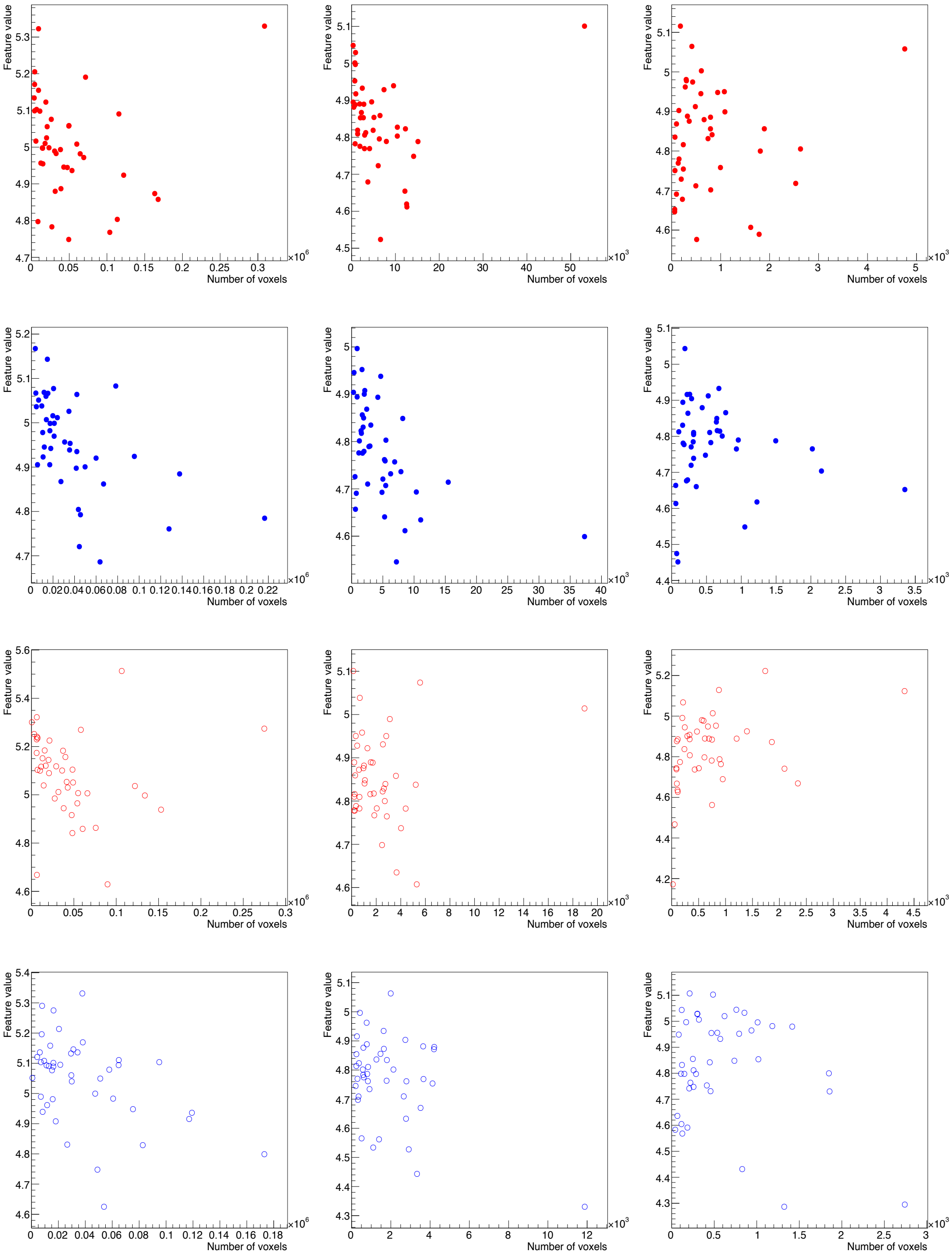

Figure SF8: Values of the feature GLRLM RunEntropy (each marker represents a patient) for tumour (red) and muscle (blue) in images with 2mm (solid markers, top two rows) and 5mm (hollow markers, bottom two rows) slice thickness. A comparison is presented for the values computed with a voxel size of (0.5,0.5,0.5)mm on the left column, vs the original voxel sizes on the middle column, and (2.0,2.0,2.0)mm on the right column.

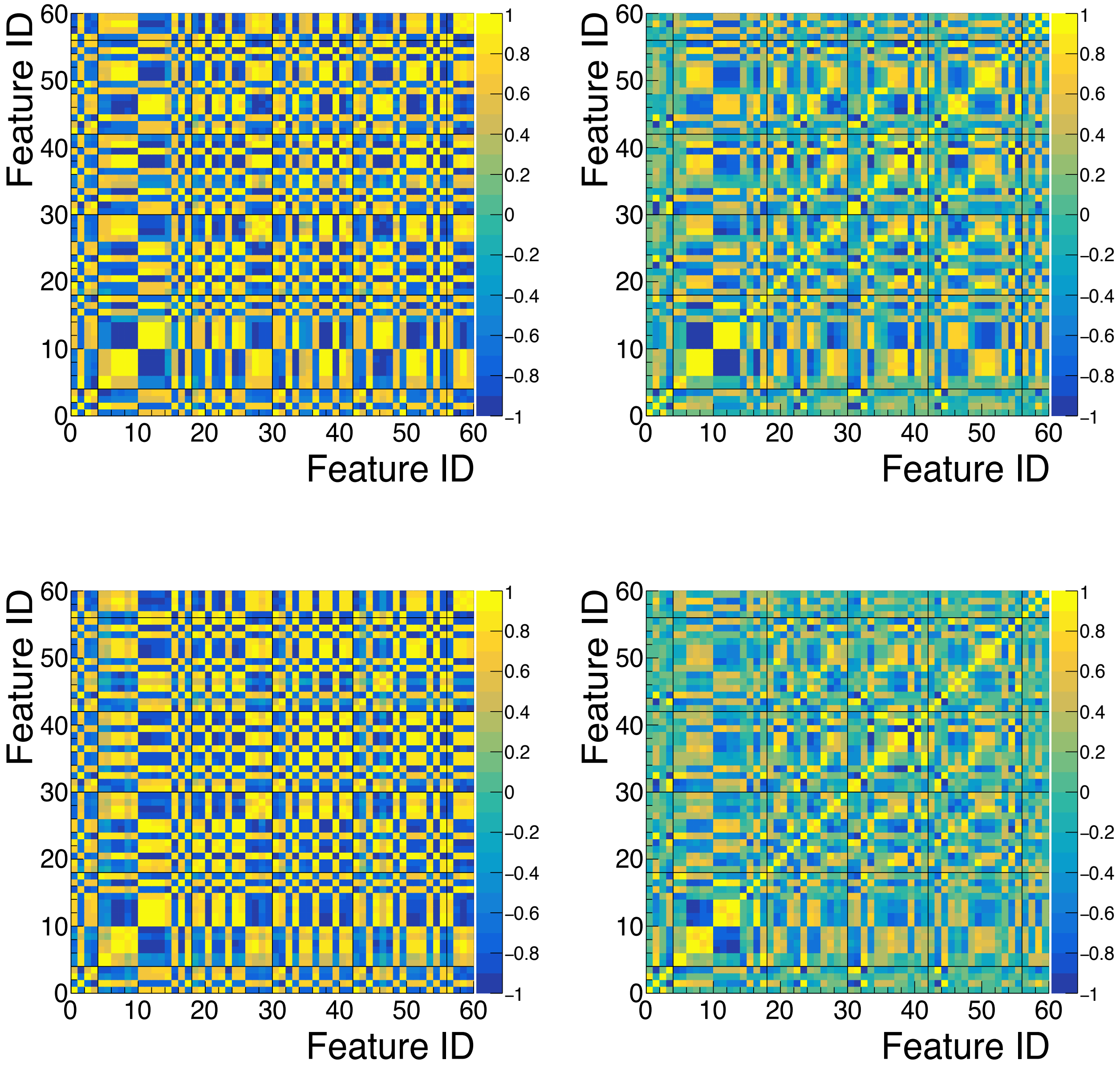

Figure SF9: Matrices of Spearman's rank correlation coefficient showing pairwise correlations between the features before (left column) and after (right column) correcting the dependencies of features with the number of voxels for the **tumour** VOIs calculated in CT images with reconstructed slice thickness of 2mm (top row) and 5mm (bottom row) and the original voxel size (i.e. no resampling). Feature ID in this Figure follows the one in Supplementary Table ST3.

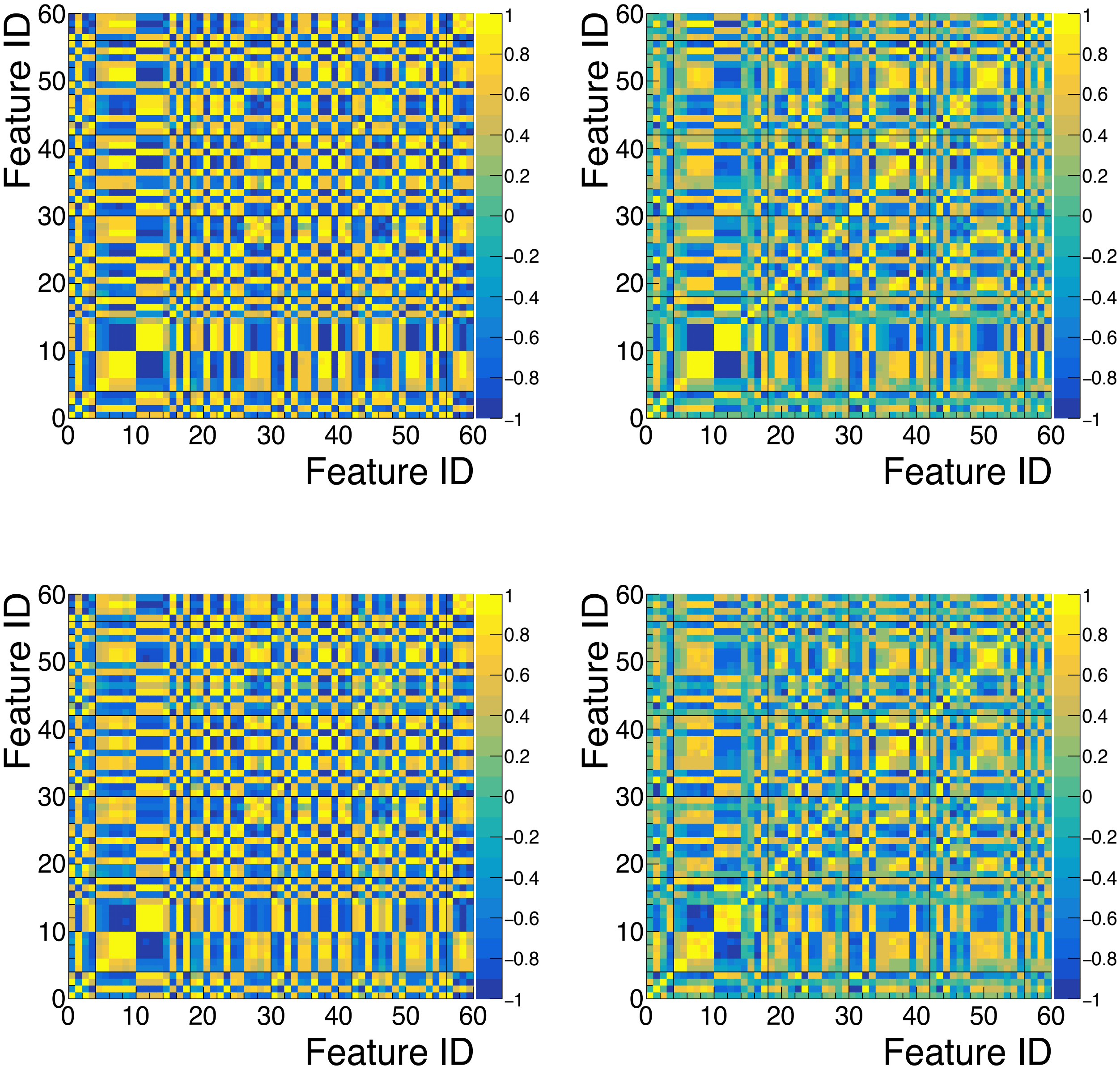

Figure SF10: Matrices of Spearmans rank correlation coefficient showing pairwise correlations between the features before (left column) and after (right column) correcting the dependencies of features with the number of voxels for the **muscle** VOIs calculated in CT images with reconstructed slice thickness of 2mm (top row) and 5mm (bottom row)and the original voxel size (i.e. no resampling). Feature ID in this Figure follows the one in Supplementary Table ST3.

Feature	ICC tumour (2mm)	ICC muscle (2mm)	ICC tumour (5mm)	ICC muscle (5mm)
firstorder Entropy	0.930	0.966	0.877	0.961
firstorder Uniformity	0.409	0.421	0.430	0.435
glcm Autocorrelation	0.122	0.122	0.122	0.122
glcm ClusterProminence	0.022	0.022	0.022	0.022
glcm ClusterShade	0.049	0.049	0.049	0.049
glcm ClusterTendency	0.121	0.121	0.121	0.121
glcm Contrast	0.121	0.121	0.121	0.121
glcm DifferenceAverage	0.387	0.388	0.388	0.387
glcm DifferenceVariance	0.121	0.121	0.121	0.121
glcm Id	0.867	0.870	0.892	0.897
glcm Idm	0.739	0.745	0.787	0.797
glcm Imc1	0.542	0.496	0.590	0.485
glcm Imc2	0.700	0.661	0.718	0.670
glcm InverseVariance	0.668	0.654	0.495	0.542
glcm JointAverage	0.388	0.388	0.388	0.388
glcm JointEnergy	0.117	0.133	0.160	0.161
glcm JointEntropy	0.154	0.161	0.408	0.391
glcm MCC	0.843	0.829	0.873	0.822
glcm MaximumProbability	0.106	0.119	0.185	0.191
glcm SumAverage	0.388	0.388	0.388	0.388
glcm SumEntropy	0.764	0.801	0.781	0.893
glcm SumSquares	0.121	0.121	0.121	0.121
gldm DependenceEntropy	0.920	0.899	0.979	0.962
gldm DependenceNonUniformity	0.626	0.627	0.622	0.617
gldm DependenceNonUniformityNormalized	0.758	0.808	0.856	0.849
gldm DependenceVariance	0.310	0.300	0.374	0.393
gldm GrayLevelNonUniformity	0.419	0.421	0.434	0.428
gldm GrayLevelVariance	0.121	0.121	0.121	0.121
gldm HighGrayLevelEmphasis	0.122	0.122	0.122	0.122
gldm LargeDependenceEmphasis	0.221	0.229	0.280	0.287
gldm LargeDependenceHighGrayLevelEmphasis	0.511	0.513	0.545	0.541
gldm LargeDependenceLowGrayLevelEmphasis	0.038	0.038	0.046	0.053
gldm LowGrayLevelEmphasis	0.305	0.317	0.374	0.277
gldm SmallDependenceEmphasis	0.836	0.851	0.854	0.842
gldm SmallDependenceHighGrayLevelEmphasis	0.095	0.085	0.068	0.082
gldm SmallDependenceLowGrayLevelEmphasis	0.664	0.745	0.694	0.529
glrlm GrayLevelNonUniformity	0.558	0.556	0.620	0.608
glrlm GrayLevelNonUniformityNormalized	0.459	0.487	0.500	0.540
glrlm GrayLevelVariance	0.121	0.121	0.121	0.121
glrlm HighGrayLevelRunEmphasis	0.122	0.122	0.122	0.122
glrlm LongRunEmphasis	0.197	0.211	0.164	0.196
glrlm LongRunHighGrayLevelEmphasis	0.138	0.141	0.153	0.148
glrlm LongRunLowGrayLevelEmphasis	0.061	0.106	0.059	0.047
glrlm LowGrayLevelRunEmphasis	0.302	0.310	0.367	0.269
glrlm RunEntropy	0.721	0.633	0.656	0.646
glrlm RunLengthNonUniformity	0.898	0.912	0.842	0.884
glrlm RunLengthNonUniformityNormalized	0.599	0.603	0.658	0.670

Table ST3: Values of ICC(3,1) for muscle and tumour, in 2mm and 5mm slices, independently. Those features with ICC(3,1)<0.9 are considered correlated, except the ones shown in blue, for which only one value appears just below 0.9 in the four cases, and therefore are treated as fluctuations.

Feature	ICC tumour (2mm)	ICC muscle (2mm)	ICC tumour (5mm)	ICC muscle (5mm)
glrlm RunPercentage	0.472	0.480	0.538	0.555
glrlm RunVariance	0.169	0.184	0.139	0.171
glrlm ShortRunEmphasis	0.452	0.460	0.512	0.531
glrlm ShortRunHighGrayLevelEmphasis	0.119	0.118	0.117	0.118
glrlm ShortRunLowGrayLevelEmphasis	0.321	0.334	0.389	0.295
glszm GrayLevelNonUniformity	0.855	0.851	0.767	0.779
glszm GrayLevelNonUniformityNormalized	0.275	0.312	0.271	0.314
glszm GrayLevelVariance	0.117	0.117	0.115	0.117
glszm HighGrayLevelZoneEmphasis	0.115	0.118	0.118	0.118
glszm LargeAreaEmphasis	0.060	0.051	0.091	0.072
glszm LargeAreaHighGrayLevelEmphasis	0.226	0.185	0.412	0.282
glszm LargeAreaLowGrayLevelEmphasis	0.016	0.013	0.022	0.021
glszm LowGrayLevelZoneEmphasis	0.161	0.165	0.193	0.155
glszm SizeZoneNonUniformity	0.377	0.397	0.341	0.354
glszm SizeZoneNonUniformityNormalized	0.664	0.774	0.847	0.857
glszm SmallAreaEmphasis	0.539	0.726	0.779	0.830
glszm SmallAreaHighGrayLevelEmphasis	0.100	0.097	0.089	0.094
glszm SmallAreaLowGrayLevelEmphasis	0.146	0.187	0.327	0.242
glszm ZoneEntropy	0.818	0.717	0.837	0.776
glszm ZonePercentage	0.786	0.796	0.799	0.796
glszm ZoneVariance	0.060	0.051	0.092	0.072
ngtdm Busyness	0.125	0.129	0.125	0.130
ngtdm Coarseness	0.986048	0.987755	0.873	0.950
ngtdm Complexity	0.051	0.048	0.052	0.051
ngtdm Contrast	0.223	0.242	0.084	0.165
ngtdm Strength	0.180	0.176	0.218	0.190

Table ST4: Values of ICC(3,1) for muscle and tumour, in 2mm and 5mm slices, independently. Those features with ICC(3,1)$<$ 0.9 are considered correlated, except the ones shown in blue, for which only one value appears just below 0.9 in the four cases, and therefore are treated as fluctuations.

Feature	ID	r_S tumour (2mm)	r_S muscle (2mm)	r_S tumour (5mm)	r_S muscle (5mm)
firstorder Energy	0	0.888	0.925	0.871	0.925
firstorder Entropy	1	-0.671	-0.786	-0.648	-0.639
firstorder TotalEnergy	2	0.888	0.925	0.871	0.924
firstorder Uniformity	3	0.639	0.791	0.621	0.621
glcm ClusterProminence	4	-0.662	-0.640	-0.677	-0.615
glcm ClusterTendency	5	-0.591	-0.710	-0.613	-0.636
glcm Contrast	6	-0.710	-0.725	-0.772	-0.646
glcm DifferenceAverage	7	-0.701	-0.727	-0.773	-0.653
glcm DifferenceEntropy	8	-0.648	-0.658	-0.615	-0.561
glcm DifferenceVariance	9	-0.715	-0.715	-0.711	-0.637
glcm Id	10	0.693	0.714	0.744	0.649
glcm Idm	11	0.682	0.709	0.738	0.651
glcm Idmn	12	0.708	0.724	0.772	0.645
glcm Idn	13	0.693	0.726	0.770	0.654
glcm Imc1	14	0.759	0.809	0.832	0.886
glcm Imc2	15	-0.812	-0.861	-0.853	-0.913
glcm InverseVariance	16	0.669	0.709	0.726	0.652
glcm SumSquares	17	-0.703	-0.773	-0.693	-0.672
gldm DependenceEntropy	18	0.729	0.775	0.757	0.814
gldm DependenceNonUniformity	19	0.994	0.985	0.987	0.981
gldm DependenceNonUniformityNormalized	20	-0.864	-0.820	-0.851	-0.828
gldm DependenceVariance	21	0.851	0.783	0.835	0.791
gldm GrayLevelNonUniformity	22	0.995	0.996	0.993	0.989
gldm GrayLevelVariance	23	-0.707	-0.819	-0.713	-0.705
gldm LargeDependenceEmphasis	24	0.869	0.811	0.851	0.824
gldm LargeDependenceHighGrayLevelEmphasis	25	0.731	0.676	0.710	0.611
gldm LowGrayLevelEmphasis	26	-0.713	-0.704	-0.751	-0.666
gldm SmallDependenceEmphasis	27	-0.865	-0.818	-0.856	-0.803
gldm SmallDependenceHighGrayLevelEmphasis	28	-0.783	-0.598	-0.767	-0.743
gldm SmallDependenceLowGrayLevelEmphasis	29	-0.833	-0.799	-0.831	-0.788
glrlm GrayLevelNonUniformity	30	0.996	0.996	0.993	0.989
glrlm GrayLevelNonUniformityNormalized	31	0.636	0.788	0.615	0.624
glrlm GrayLevelVariance	32	-0.703	-0.819	-0.714	-0.706
glrlm LongRunEmphasis	33	0.871	0.814	0.821	0.790
glrlm LongRunLowGrayLevelEmphasis	34	-0.644	-0.675	-0.719	-0.589
glrlm LowGrayLevelRunEmphasis	35	-0.713	-0.707	-0.753	-0.661
glrlm RunLengthNonUniformity	36	0.999	0.998	0.999	0.997
glrlm RunLengthNonUniformityNormalized	37	-0.863	-0.813	-0.813	-0.792
glrlm RunPercentage	38	-0.872	-0.811	-0.828	-0.793
glrlm RunVariance	39	0.863	0.818	0.822	0.797
glrlm ShortRunEmphasis	40	-0.865	-0.808	-0.818	-0.790
glrlm ShortRunLowGrayLevelEmphasis	41	-0.717	-0.704	-0.766	-0.679
glszm GrayLevelNonUniformity	42	0.993	0.986	0.991	0.985
glszm GrayLevelNonUniformityNormalized	43	0.470	0.683	0.533	0.531
glszm GrayLevelVariance	44	-0.647	-0.733	-0.711	-0.643
glszm LargeAreaEmphasis	45	0.893	0.852	0.862	0.861
glszm LargeAreaHighGrayLevelEmphasis	46	0.880	0.814	0.832	0.794
glszm LargeAreaLowGrayLevelEmphasis	47	0.627	0.661	0.595	0.624
glszm LowGrayLevelZoneEmphasis	48	-0.717	-0.721	-0.776	-0.681
glszm SizeZoneNonUniformity	49	0.972	0.937	0.949	0.891
glszm SizeZoneNonUniformityNormalized	50	-0.815	-0.754	-0.831	-0.739
glszm SmallAreaEmphasis	51	-0.798	-0.752	-0.824	-0.737
glszm SmallAreaLowGrayLevelEmphasis	52	-0.764	-0.765	-0.751	-0.691
glszm ZoneEntropy	53	0.912	0.860	0.913	0.909
glszm ZonePercentage	54	-0.881	-0.830	-0.859	-0.836
glszm ZoneVariance	55	0.895	0.856	0.862	0.868
ngtdm Busyness	56	0.988	0.957	0.974	0.967
ngtdm Coarseness	57	-0.995	-0.995	-0.993	-0.991
ngtdm Contrast	58	-0.858	-0.851	-0.851	-0.783
ngtdm Strength	59	-0.988	-0.960	-0.980	-0.965

Table ST5: Median values of Spearman's rank correlation coefficient (r_S), across different non-extreme voxel size resampling and interpolation method, for features found correlated ($r_S > 0.5$) with the number of voxels. High correlations are shown in red ($r_S > 0.75$) and medium in blue ($0.75 > r_S > 0.5$)

Feature	O	(1,1,1)			(μ_x, μ_y, Z)			(μ_x, μ_y, μ_z)			(1.25,1.25,1.25)		
		L	S	W	L	S	W	L	S	W	L	S	W
shape Elongation	0.18	0.20	0.20	0.20	0.20	0.20	0.20	0.18	0.18	0.18	0.18	0.18	0.18
shape Flatness	0.19	0.20	0.20	0.20	0.19	0.19	0.19	0.21	0.21	0.21	0.19	0.19	0.19
shape LeastAxisLength	0.14	0.12	0.12	0.12	0.13	0.13	0.13	0.12	0.12	0.12	0.12	0.12	0.12
shape MajorAxisLength	0.18	0.18	0.18	0.18	0.19	0.19	0.19	0.18	0.18	0.18	0.17	0.17	0.17
shape Maximum2DDiameterColumn	0.15	0.14	0.14	0.14	0.15	0.15	0.15	0.14	0.14	0.14	0.14	0.14	0.14
shape Maximum2DDiameterRow	0.14	0.14	0.14	0.14	0.14	0.14	0.14	0.14	0.14	0.14	0.14	0.14	0.14
shape Maximum2DDiameterSlice	0.10	0.09	0.09	0.09	0.08	0.08	0.08	0.11	0.11	0.11	0.08	0.08	0.08
shape Maximum3DDiameter	0.15	0.14	0.14	0.14	0.15	0.15	0.15	0.15	0.15	0.15	0.15	0.15	0.15
shape MeshVolume	0.21	0.21	0.21	0.21	0.21	0.21	0.21	0.21	0.21	0.21	0.20	0.20	0.20
shape MinorAxisLength	0.07	0.08	0.08	0.08	0.07	0.07	0.07	0.07	0.07	0.07	0.08	0.08	0.08
shape Sphericity	0.14	0.16	0.16	0.16	0.14	0.14	0.14	0.07	0.07	0.07	0.13	0.13	0.13
shape SurfaceArea	0.24	0.23	0.23	0.23	0.25	0.25	0.25	0.16	0.16	0.16	0.24	0.24	0.24
shape SurfaceVolumeRatio	0.14	0.14	0.14	0.14	0.13	0.13	0.13	0.06	0.06	0.06	0.12	0.12	0.12
shape VoxelVolume	0.20	0.21	0.21	0.21	0.20	0.20	0.20	0.21	0.21	0.21	0.20	0.20	0.20
firstorder 10Percentile	0.28	0.19	0.23	0.19	0.23	0.27	0.26	0.23	0.29	0.25	0.21	0.23	0.19
firstorder 90Percentile	0.07	0.06	0.06	0.06	0.06	0.07	0.07	0.06	0.06	0.06	0.05	0.06	0.06
firstorder Energy	0.63	0.24	0.24	0.24	0.62	0.63	0.63	0.26	0.24	0.24	0.23	0.23	0.23
firstorder Entropy	0.02	0.03	0.02	0.03	0.03	0.03	0.03	0.02	0.02	0.03	0.03	0.02	0.02
firstorder InterquartileRange	0.23	0.25	0.23	0.20	0.22	0.22	0.22	0.26	0.22	0.22	0.26	0.23	0.21
firstorder Kurtosis	0.07	0.10	0.07	0.08	0.07	0.07	0.07	0.09	0.06	0.06	0.10	0.07	0.08
firstorder Maximum	0.16	0.13	0.15	0.15	0.16	0.18	0.18	0.12	0.14	0.13	0.15	0.14	0.14
firstorder MeanAbsoluteDeviation	0.21	0.22	0.22	0.20	0.22	0.22	0.21	0.24	0.23	0.21	0.25	0.22	0.20
firstorder Mean	0.04	0.04	0.04	0.04	0.04	0.04	0.04	0.03	0.03	0.03	0.04	0.03	0.03
firstorder Median	0.04	0.03	0.04	0.04	0.04	0.05	0.05	0.04	0.04	0.04	0.04	0.04	0.04
firstorder Minimum	1.05	0.84	1.08	1.11	0.86	1.04	1.02	0.86	1.12	1.06	0.93	0.99	0.94
firstorder Range	0.28	0.22	0.26	0.22	0.29	0.29	0.30	0.26	0.24	0.23	0.27	0.25	0.24
firstorder RobustMeanAbsoluteDeviation	0.21	0.24	0.22	0.21	0.22	0.22	0.22	0.23	0.22	0.21	0.26	0.23	0.21
firstorder RootMeanSquared	0.03	0.04	0.03	0.03	0.03	0.03	0.03	0.04	0.04	0.04	0.04	0.03	0.03
firstorder Skewness	0.92	1.22	1.06	1.35	1.24	0.96	1.05	1.07	1.24	1.16	1.21	1.25	1.19
firstorder TotalEnergy	0.24	0.24	0.24	0.24	0.22	0.22	0.22	0.26	0.24	0.24	0.23	0.23	0.23
firstorder Uniformity	0.09	0.10	0.10	0.10	0.10	0.09	0.08	0.09	0.09	0.11	0.10	0.08	0.08
firstorder Variance	0.38	0.40	0.37	0.35	0.40	0.39	0.38	0.43	0.41	0.38	0.42	0.38	0.36
glcm Autocorrelation	0.10	0.16	0.18	0.17	0.12	0.12	0.11	0.19	0.18	0.17	0.24	0.22	0.20
glcm ClusterProminence	0.23	0.31	0.28	0.30	0.25	0.27	0.27	0.30	0.33	0.36	0.44	0.35	0.31
glcm ClusterShade	1.02	1.07	1.29	1.44	1.05	0.98	1.18	1.09	1.26	1.13	1.80	1.58	1.83
glcm ClusterTendency	0.12	0.18	0.16	0.15	0.19	0.15	0.15	0.18	0.24	0.21	0.19	0.17	0.16
glcm Contrast	0.41	0.24	0.19	0.21	0.47	0.47	0.44	0.21	0.18	0.19	0.21	0.17	0.19
glcm Correlation	0.40	0.13	0.11	0.12	0.37	0.39	0.39	0.41	0.57	0.48	0.22	0.25	0.26
glcm DifferenceAverage	0.20	0.12	0.10	0.11	0.21	0.21	0.20	0.11	0.10	0.09	0.10	0.09	0.09
glcm DifferenceEntropy	0.06	0.04	0.03	0.04	0.05	0.06	0.06	0.04	0.03	0.03	0.04	0.03	0.04
glcm DifferenceVariance	0.37	0.20	0.19	0.22	0.40	0.43	0.44	0.22	0.18	0.18	0.19	0.17	0.20
glcm Id	0.11	0.08	0.06	0.06	0.12	0.11	0.11	0.08	0.06	0.06	0.08	0.05	0.06
glcm Idm	0.15	0.12	0.10	0.10	0.16	0.15	0.15	0.13	0.09	0.08	0.13	0.09	0.10
glcm Idmn	0.01	0.00	0.00	0.00	0.01	0.01	0.01	0.01	0.01	0.01	0.01	0.01	0.01
glcm Idn	0.02	0.01	0.01	0.01	0.02	0.02	0.02	0.01	0.01	0.01	0.01	0.01	0.01
glcm Imc1	0.56	0.30	0.32	0.32	0.56	0.68	0.66	0.29	0.25	0.22	0.31	0.28	0.31
glcm Imc2	0.15	0.05	0.07	0.07	0.13	0.16	0.16	0.06	0.07	0.06	0.07	0.07	0.06
glcm InverseVariance	0.14	0.12	0.08	0.09	0.16	0.15	0.15	0.11	0.10	0.09	0.11	0.09	0.09
glcm JointAverage	0.05	0.09	0.09	0.08	0.06	0.07	0.05	0.11	0.09	0.09	0.12	0.12	0.10
glcm JointEnergy	0.20	0.23	0.15	0.18	0.20	0.21	0.20	0.19	0.17	0.17	0.22	0.20	0.19
glcm JointEntropy	0.03	0.03	0.03	0.03	0.04	0.03	0.03	0.03	0.03	0.03	0.03	0.03	0.03
glcm MCC	0.18	0.11	0.15	0.15	0.20	0.21	0.20	0.25	0.21	0.18	0.16	0.16	0.15
glcm MaximumProbability	0.24	0.21	0.15	0.21	0.22	0.21	0.20	0.15	0.15	0.13	0.21	0.18	0.17
glcm SumAverage	0.05	0.09	0.09	0.08	0.06	0.07	0.05	0.11	0.09	0.09	0.12	0.12	0.10
glcm SumEntropy	0.02	0.02	0.02	0.02	0.02	0.03	0.02	0.03	0.03	0.03	0.03	0.02	0.02
glcm SumSquares	0.18	0.21	0.16	0.19	0.22	0.18	0.17	0.18	0.18	0.20	0.22	0.15	0.16

Table ST6: Median values of the relative difference between the values of the features computed in images with 2mm and 5mm slice thickness (Δ_r), across different non-extreme voxel size resampling and interpolation method, before correcting for the dependence with the number of voxels. Values $\Delta_r \geq 0.5$ are shown in red.

Feature	O	(1,1,1)			(μ_x, μ_y, Z)			(μ_x, μ_y, μ_z)			(1.25,1.25,1.25)		
		L	S	W	L	S	W	L	S	W	L	S	W
gldm DependenceEntropy	0.03	0.02	0.03	0.03	0.03	0.03	0.04	0.02	0.02	0.02	0.03	0.03	0.02
gldm DependenceNonUniformity	0.54	0.27	0.24	0.23	0.54	0.55	0.52	0.20	0.19	0.20	0.24	0.24	0.25
gldm DependenceNonUniformityNormalized	0.24	0.14	0.11	0.10	0.24	0.25	0.26	0.11	0.10	0.10	0.12	0.10	0.10
gldm DependenceVariance	0.27	0.41	0.26	0.26	0.31	0.30	0.31	0.22	0.14	0.12	0.23	0.28	0.26
gldm GrayLevelNonUniformity	0.65	0.26	0.28	0.26	0.65	0.64	0.65	0.25	0.25	0.26	0.25	0.25	0.22
gldm GrayLevelVariance	0.19	0.20	0.16	0.16	0.19	0.19	0.19	0.17	0.17	0.19	0.20	0.14	0.17
gldm HighGrayLevelEmphasis	0.06	0.14	0.18	0.15	0.09	0.12	0.10	0.15	0.14	0.15	0.20	0.19	0.17
gldm LargeDependenceEmphasis	0.29	0.31	0.25	0.22	0.29	0.30	0.33	0.18	0.14	0.16	0.25	0.20	0.21
gldm LargeDependenceHighGrayLevelEmphasis	0.24	0.56	0.41	0.42	0.26	0.25	0.22	0.40	0.32	0.21	0.44	0.44	0.30
gldm LargeDependenceLowGrayLevelEmphasis	0.23	0.20	0.24	0.24	0.28	0.29	0.31	0.26	0.24	0.17	0.35	0.29	0.27
gldm LowGrayLevelEmphasis	0.31	0.24	0.28	0.20	0.29	0.26	0.26	0.31	0.27	0.24	0.32	0.24	0.21
gldm SmallDependenceEmphasis	0.20	0.16	0.14	0.13	0.25	0.25	0.27	0.11	0.10	0.08	0.15	0.12	0.11
gldm SmallDependenceHighGrayLevelEmphasis	0.33	0.13	0.09	0.11	0.34	0.33	0.32	0.18	0.15	0.14	0.16	0.16	0.14
gldm SmallDependenceLowGrayLevelEmphasis	0.45	0.33	0.42	0.36	0.40	0.33	0.41	0.34	0.31	0.29	0.40	0.33	0.25
glrlm GrayLevelNonUniformity	0.64	0.25	0.26	0.25	0.64	0.64	0.64	0.26	0.25	0.25	0.25	0.20	0.23
glrlm GrayLevelNonUniformityNormalized	0.08	0.10	0.09	0.10	0.10	0.09	0.08	0.09	0.09	0.11	0.10	0.07	0.08
glrlm GrayLevelVariance	0.19	0.20	0.15	0.16	0.19	0.18	0.19	0.17	0.16	0.19	0.19	0.14	0.17
glrlm HighGrayLevelRunEmphasis	0.06	0.13	0.18	0.15	0.09	0.12	0.10	0.15	0.14	0.14	0.20	0.19	0.18
glrlm LongRunEmphasis	0.04	0.08	0.05	0.05	0.04	0.04	0.04	0.02	0.02	0.02	0.05	0.03	0.03
glrlm LongRunHighGrayLevelEmphasis	0.08	0.20	0.23	0.21	0.10	0.11	0.09	0.19	0.16	0.16	0.27	0.22	0.19
glrlm LongRunLowGrayLevelEmphasis	0.24	0.22	0.23	0.22	0.27	0.22	0.20	0.29	0.25	0.23	0.32	0.20	0.22
glrlm LowGrayLevelRunEmphasis	0.31	0.23	0.28	0.20	0.29	0.27	0.26	0.30	0.28	0.23	0.33	0.24	0.22
glrlm RunEntropy	0.02	0.02	0.02	0.02	0.02	0.02	0.02	0.02	0.02	0.02	0.02	0.02	0.02
glrlm RunLengthNonUniformity	0.59	0.22	0.21	0.20	0.59	0.59	0.59	0.22	0.22	0.22	0.22	0.21	0.21
glrlm RunLengthNonUniformityNormalized	0.03	0.02	0.01	0.02	0.03	0.03	0.03	0.01	0.01	0.01	0.02	0.01	0.01
glrlm RunPercentage	0.01	0.02	0.01	0.01	0.01	0.01	0.02	0.01	0.01	0.01	0.01	0.01	0.01
glrlm RunVariance	0.29	0.65	0.42	0.41	0.27	0.29	0.30	0.18	0.16	0.16	0.45	0.34	0.32
glrlm ShortRunEmphasis	0.01	0.01	0.01	0.01	0.01	0.01	0.01	0.01	0.00	0.01	0.01	0.01	0.01
glrlm ShortRunHighGrayLevelEmphasis	0.07	0.12	0.15	0.14	0.08	0.13	0.12	0.14	0.14	0.14	0.21	0.18	0.18
glrlm ShortRunLowGrayLevelEmphasis	0.33	0.24	0.29	0.21	0.29	0.29	0.28	0.31	0.28	0.24	0.33	0.25	0.20
glszm GrayLevelNonUniformity	0.55	0.29	0.26	0.25	0.53	0.55	0.55	0.25	0.22	0.22	0.27	0.26	0.26
glszm GrayLevelNonUniformityNormalized	0.05	0.07	0.07	0.07	0.05	0.06	0.06	0.07	0.06	0.07	0.06	0.06	0.07
glszm GrayLevelVariance	0.12	0.11	0.11	0.12	0.13	0.12	0.12	0.13	0.12	0.14	0.13	0.13	0.15
glszm HighGrayLevelZoneEmphasis	0.07	0.13	0.16	0.12	0.09	0.11	0.09	0.12	0.12	0.13	0.19	0.19	0.16
glszm LargeAreaEmphasis	0.56	1.46	0.59	0.48	0.54	0.51	0.55	0.52	0.26	0.30	0.42	0.40	0.40
glszm LargeAreaHighGrayLevelEmphasis	0.52	1.58	0.80	0.54	0.56	0.49	0.51	0.61	0.39	0.33	0.59	0.52	0.46
glszm LargeAreaLowGrayLevelEmphasis	0.38	0.67	0.42	0.37	0.50	0.44	0.49	0.38	0.32	0.27	0.51	0.35	0.40
glszm LowGrayLevelZoneEmphasis	0.27	0.17	0.27	0.21	0.23	0.23	0.23	0.29	0.31	0.21	0.29	0.31	0.26
glszm SizeZoneNonUniformity	0.45	0.27	0.28	0.26	0.47	0.47	0.43	0.25	0.24	0.24	0.30	0.25	0.28
glszm SizeZoneNonUniformityNormalized	0.14	0.07	0.11	0.09	0.17	0.17	0.19	0.10	0.11	0.08	0.11	0.09	0.10
glszm SmallAreaEmphasis	0.07	0.04	0.06	0.04	0.08	0.08	0.09	0.05	0.05	0.04	0.06	0.04	0.05
glszm SmallAreaHighGrayLevelEmphasis	0.10	0.13	0.11	0.13	0.14	0.16	0.15	0.12	0.11	0.12	0.13	0.16	0.12
glszm SmallAreaLowGrayLevelEmphasis	0.26	0.25	0.40	0.23	0.21	0.28	0.31	0.31	0.30	0.28	0.30	0.36	0.31
glszm ZoneEntropy	0.04	0.02	0.03	0.03	0.05	0.05	0.05	0.02	0.03	0.02	0.03	0.03	0.03
glszm ZonePercentage	0.21	0.19	0.11	0.13	0.25	0.23	0.24	0.10	0.09	0.08	0.14	0.10	0.12
glszm ZoneVariance	0.69	2.04	0.74	0.60	0.74	0.66	0.69	0.59	0.31	0.41	0.58	0.59	0.54
ngtdm Busyness	0.52	0.30	0.24	0.20	0.49	0.54	0.53	0.30	0.23	0.21	0.24	0.20	0.19
ngtdm Coarseness	1.26	0.25	0.24	0.22	1.30	1.33	1.31	0.34	0.31	0.27	0.26	0.26	0.24
ngtdm Complexity	0.14	0.15	0.11	0.11	0.14	0.17	0.14	0.18	0.13	0.12	0.17	0.11	0.11
ngtdm Contrast	0.51	0.28	0.24	0.26	0.54	0.37	0.46	0.26	0.24	0.20	0.22	0.22	0.22
ngtdm Strength	1.09	0.29	0.23	0.26	1.08	1.22	1.13	0.38	0.28	0.27	0.32	0.21	0.22

Table ST7: Median values of the relative difference between the values of the features computed in images with 2mm and 5mm slice thickness (Δ_r), across different non-extreme voxel size resampling and interpolation method, before correcting for the dependence with the number of voxels. Values $\Delta_r \geq 0.5$ are shown in red.

Feature	O	(1,1,1)			(μ_x, μ_y, Z)			(μ_x, μ_y, μ_z)			(1.25,1.25,1.25)		
		L	S	W	L	S	W	L	S	W	L	S	W
shape Elongation	0.18	0.20	0.20	0.20	0.20	0.20	0.20	0.18	0.18	0.18	0.18	0.18	0.18
shape Flatness	0.19	0.20	0.20	0.20	0.19	0.19	0.19	0.21	0.21	0.21	0.19	0.19	0.19
shape LeastAxisLength	0.14	0.12	0.12	0.12	0.13	0.13	0.13	0.12	0.12	0.12	0.12	0.12	0.12
shape MajorAxisLength	0.18	0.18	0.18	0.18	0.19	0.19	0.19	0.18	0.18	0.18	0.17	0.17	0.17
shape Maximum2DDiameterColumn	0.15	0.14	0.14	0.14	0.15	0.15	0.15	0.14	0.14	0.14	0.14	0.14	0.14
shape Maximum2DDiameterRow	0.14	0.14	0.14	0.14	0.14	0.14	0.14	0.14	0.14	0.14	0.14	0.14	0.14
shape Maximum2DDiameterSlice	0.10	0.09	0.09	0.09	0.08	0.08	0.08	0.11	0.11	0.11	0.08	0.08	0.08
shape Maximum3DDiameter	0.15	0.14	0.14	0.14	0.15	0.15	0.15	0.15	0.15	0.15	0.15	0.15	0.15
shape MeshVolume	0.21	0.21	0.21	0.21	0.21	0.21	0.21	0.21	0.21	0.21	0.20	0.20	0.20
shape MinorAxisLength	0.07	0.08	0.08	0.08	0.07	0.07	0.07	0.07	0.07	0.07	0.08	0.08	0.08
shape Sphericity	0.14	0.16	0.16	0.16	0.14	0.14	0.14	0.07	0.07	0.07	0.13	0.13	0.13
shape SurfaceArea	0.24	0.23	0.23	0.23	0.25	0.25	0.25	0.16	0.16	0.16	0.24	0.24	0.24
shape SurfaceVolumeRatio	0.14	0.14	0.14	0.14	0.13	0.13	0.13	0.06	0.06	0.06	0.12	0.12	0.12
shape VoxelVolume	0.20	0.21	0.21	0.21	0.20	0.20	0.20	0.21	0.21	0.21	0.20	0.20	0.20
firstorder 10Percentile	0.28	0.19	0.23	0.19	0.23	0.27	0.26	0.23	0.29	0.25	0.21	0.23	0.19
firstorder 90Percentile	0.07	0.06	0.06	0.06	0.06	0.07	0.07	0.06	0.06	0.06	0.05	0.06	0.06
firstorder Energy	0.08	0.08	0.08	0.08	0.09	0.11	0.11	0.09	0.09	0.09	0.08	0.08	0.09
firstorder Entropy	0.02	0.03	0.02	0.03	0.03	0.03	0.03	0.02	0.02	0.03	0.03	0.02	0.02
firstorder InterquartileRange	0.23	0.25	0.23	0.20	0.22	0.22	0.22	0.26	0.22	0.22	0.26	0.23	0.21
firstorder Kurtosis	0.07	0.10	0.07	0.08	0.07	0.07	0.07	0.09	0.06	0.06	0.10	0.07	0.08
firstorder Maximum	0.16	0.13	0.15	0.15	0.16	0.18	0.18	0.12	0.14	0.13	0.15	0.14	0.14
firstorder MeanAbsoluteDeviation	0.21	0.22	0.22	0.20	0.22	0.22	0.21	0.24	0.23	0.21	0.25	0.22	0.20
firstorder Mean	0.04	0.04	0.04	0.04	0.04	0.04	0.04	0.03	0.03	0.03	0.04	0.03	0.03
firstorder Median	0.04	0.03	0.04	0.04	0.04	0.05	0.05	0.04	0.04	0.04	0.04	0.04	0.04
firstorder Minimum	1.05	0.84	1.08	1.11	0.86	1.04	1.02	0.86	1.12	1.06	0.93	0.99	0.94
firstorder Range	0.28	0.22	0.26	0.22	0.29	0.29	0.30	0.26	0.24	0.23	0.27	0.25	0.24
firstorder RobustMeanAbsoluteDeviation	0.21	0.24	0.22	0.21	0.22	0.22	0.22	0.23	0.22	0.21	0.26	0.23	0.21
firstorder RootMeanSquared	0.03	0.04	0.03	0.03	0.03	0.03	0.03	0.04	0.04	0.04	0.04	0.03	0.03
firstorder Skewness	0.92	1.22	1.06	1.35	1.24	0.96	1.05	1.07	1.24	1.16	1.21	1.25	1.19
firstorder TotalEnergy	0.24	0.24	0.24	0.24	0.22	0.22	0.22	0.26	0.24	0.24	0.23	0.23	0.23
firstorder Uniformity	0.09	0.10	0.10	0.10	0.10	0.09	0.08	0.09	0.09	0.11	0.10	0.08	0.08
firstorder Variance	0.38	0.40	0.37	0.35	0.40	0.39	0.38	0.43	0.41	0.38	0.42	0.38	0.36
glcm Autocorrelation	0.10	0.16	0.18	0.17	0.12	0.12	0.11	0.19	0.18	0.17	0.24	0.22	0.20
glcm ClusterProminence	0.23	0.31	0.28	0.30	0.25	0.27	0.27	0.30	0.33	0.36	0.44	0.35	0.31
glcm ClusterShade	1.02	1.07	1.29	1.44	1.05	0.98	1.18	1.09	1.26	1.13	1.80	1.58	1.83
glcm ClusterTendency	0.12	0.18	0.16	0.15	0.19	0.15	0.15	0.18	0.24	0.21	0.19	0.17	0.16
glcm Contrast	0.41	0.24	0.19	0.21	0.47	0.47	0.44	0.21	0.18	0.19	0.21	0.17	0.19
glcm Correlation	0.40	0.13	0.11	0.12	0.37	0.39	0.39	0.41	0.57	0.48	0.22	0.25	0.26
glcm DifferenceAverage	0.20	0.12	0.10	0.11	0.21	0.21	0.20	0.11	0.10	0.09	0.10	0.09	0.09
glcm DifferenceEntropy	0.06	0.04	0.03	0.04	0.05	0.06	0.06	0.04	0.03	0.03	0.04	0.03	0.04
glcm DifferenceVariance	0.37	0.20	0.19	0.22	0.40	0.43	0.44	0.22	0.18	0.18	0.19	0.17	0.20
glcm Id	0.11	0.08	0.06	0.06	0.12	0.11	0.11	0.08	0.06	0.06	0.08	0.05	0.06
glcm Idm	0.15	0.12	0.10	0.10	0.16	0.15	0.15	0.13	0.09	0.08	0.13	0.09	0.10
glcm Idmn	0.01	0.00	0.00	0.00	0.01	0.01	0.01	0.01	0.01	0.01	0.01	0.01	0.01
glcm Idn	0.02	0.01	0.01	0.01	0.02	0.02	0.02	0.01	0.01	0.01	0.01	0.01	0.01
glcm Imc1	0.32	0.53	0.35	0.35	0.38	0.32	0.32	0.30	0.19	0.19	0.21	0.13	0.13
glcm Imc2	0.15	0.05	0.07	0.07	0.13	0.16	0.16	0.06	0.07	0.06	0.07	0.07	0.06
glcm InverseVariance	0.14	0.12	0.08	0.09	0.16	0.15	0.15	0.11	0.10	0.09	0.11	0.09	0.09
glcm JointAverage	0.05	0.09	0.09	0.08	0.06	0.07	0.05	0.11	0.09	0.09	0.12	0.12	0.10
glcm JointEnergy	0.20	0.23	0.15	0.18	0.20	0.21	0.20	0.19	0.17	0.17	0.22	0.20	0.19
glcm JointEntropy	0.03	0.03	0.03	0.03	0.04	0.03	0.03	0.03	0.03	0.03	0.03	0.03	0.03
glcm MCC	0.18	0.11	0.15	0.15	0.20	0.21	0.20	0.25	0.21	0.18	0.16	0.16	0.15
glcm MaximumProbability	0.24	0.21	0.15	0.21	0.22	0.21	0.20	0.15	0.15	0.13	0.21	0.18	0.17
glcm SumAverage	0.05	0.09	0.09	0.08	0.06	0.07	0.05	0.11	0.09	0.09	0.12	0.12	0.10
glcm SumEntropy	0.02	0.02	0.02	0.02	0.02	0.03	0.02	0.03	0.03	0.03	0.03	0.02	0.02
glcm SumSquares	0.18	0.21	0.16	0.19	0.22	0.18	0.17	0.18	0.18	0.20	0.22	0.15	0.16

Table ST8: Median values of the relative difference between the values of the features computed in images with 2mm and 5mm slice thickness (Δ_r), across different non-extreme voxel size resampling and interpolation method, after correcting certain features for the dependence with the number of voxels.Values $\Delta_r \geq 0.5$ are shown in red.

Feature	O	(1,1,1)			(μ_x, μ_y, Z)			(μ_x, μ_y, μ_z)			(1.25,1.25,1.25)		
		L	S	W	L	S	W	L	S	W	L	S	W
gldm DependenceEntropy	0.03	0.02	0.03	0.03	0.03	0.03	0.04	0.02	0.02	0.02	0.03	0.03	0.02
gldm DependenceNonUniformity	0.14	0.16	0.13	0.15	0.17	0.17	0.16	0.14	0.11	0.13	0.19	0.15	0.13
gldm DependenceNonUniformityNormalized	0.24	0.14	0.11	0.10	0.24	0.25	0.26	0.11	0.10	0.10	0.12	0.10	0.10
gldm DependenceVariance	0.27	0.41	0.26	0.26	0.31	0.30	0.31	0.22	0.14	0.12	0.23	0.28	0.26
gldm GrayLevelNonUniformity	0.09	0.10	0.08	0.11	0.11	0.10	0.11	0.08	0.09	0.10	0.12	0.09	0.10
gldm GrayLevelVariance	0.19	0.20	0.16	0.16	0.19	0.19	0.19	0.17	0.17	0.19	0.20	0.14	0.17
gldm HighGrayLevelEmphasis	0.06	0.14	0.18	0.15	0.09	0.12	0.10	0.15	0.14	0.15	0.20	0.19	0.17
gldm LargeDependenceEmphasis	0.29	0.31	0.25	0.22	0.29	0.30	0.33	0.18	0.14	0.16	0.25	0.20	0.21
gldm LargeDependenceHighGrayLevelEmphasis	0.24	0.56	0.41	0.42	0.26	0.25	0.22	0.40	0.32	0.21	0.44	0.44	0.30
gldm LargeDependenceLowGrayLevelEmphasis	0.23	0.20	0.24	0.24	0.28	0.29	0.31	0.26	0.24	0.17	0.35	0.29	0.27
gldm LowGrayLevelEmphasis	0.31	0.24	0.28	0.20	0.29	0.26	0.26	0.31	0.27	0.24	0.32	0.24	0.21
gldm SmallDependenceEmphasis	0.20	0.16	0.14	0.13	0.25	0.25	0.27	0.11	0.10	0.08	0.15	0.12	0.11
gldm SmallDependenceHighGrayLevelEmphasis	0.33	0.13	0.09	0.11	0.34	0.33	0.32	0.18	0.15	0.14	0.16	0.16	0.14
gldm SmallDependenceLowGrayLevelEmphasis	0.45	0.33	0.42	0.36	0.40	0.33	0.41	0.34	0.31	0.29	0.40	0.33	0.25
glrlm GrayLevelNonUniformity	0.08	0.08	0.08	0.08	0.09	0.09	0.11	0.08	0.08	0.10	0.10	0.07	0.09
glrlm GrayLevelNonUniformityNormalized	0.08	0.10	0.09	0.10	0.10	0.09	0.08	0.09	0.09	0.11	0.10	0.07	0.08
glrlm GrayLevelVariance	0.19	0.20	0.15	0.16	0.19	0.18	0.19	0.17	0.16	0.19	0.19	0.14	0.17
glrlm HighGrayLevelRunEmphasis	0.06	0.13	0.18	0.15	0.09	0.12	0.10	0.15	0.14	0.14	0.20	0.19	0.18
glrlm LongRunEmphasis	0.04	0.08	0.05	0.05	0.04	0.04	0.04	0.02	0.02	0.02	0.05	0.03	0.03
glrlm LongRunHighGrayLevelEmphasis	0.08	0.20	0.23	0.21	0.10	0.11	0.09	0.19	0.16	0.16	0.27	0.22	0.19
glrlm LongRunLowGrayLevelEmphasis	0.24	0.22	0.23	0.22	0.27	0.22	0.20	0.29	0.25	0.23	0.32	0.20	0.22
glrlm LowGrayLevelRunEmphasis	0.31	0.23	0.28	0.20	0.29	0.27	0.26	0.30	0.28	0.23	0.33	0.24	0.22
glrlm RunEntropy	0.02	0.02	0.02	0.02	0.02	0.02	0.02	0.02	0.02	0.02	0.02	0.02	0.02
glrlm RunLengthNonUniformity	0.02	0.03	0.03	0.03	0.05	0.05	0.05	0.02	0.02	0.02	0.04	0.03	0.03
glrlm RunLengthNonUniformityNormalized	0.03	0.02	0.01	0.02	0.03	0.03	0.03	0.01	0.01	0.01	0.02	0.01	0.01
glrlm RunPercentage	0.01	0.02	0.01	0.01	0.01	0.01	0.02	0.01	0.01	0.01	0.01	0.01	0.01
glrlm RunVariance	0.29	0.65	0.42	0.41	0.27	0.29	0.30	0.18	0.16	0.16	0.45	0.34	0.32
glrlm ShortRunEmphasis	0.01	0.01	0.01	0.01	0.01	0.01	0.01	0.01	0.00	0.01	0.01	0.01	0.01
glrlm ShortRunHighGrayLevelEmphasis	0.07	0.12	0.15	0.14	0.08	0.13	0.12	0.14	0.14	0.14	0.21	0.18	0.18
glrlm ShortRunLowGrayLevelEmphasis	0.33	0.24	0.29	0.21	0.29	0.29	0.28	0.31	0.28	0.24	0.33	0.25	0.20
glszm GrayLevelNonUniformity	0.11	0.21	0.17	0.15	0.17	0.18	0.17	0.16	0.12	0.11	0.19	0.13	0.13
glszm GrayLevelNonUniformityNormalized	0.05	0.07	0.07	0.07	0.05	0.06	0.06	0.07	0.06	0.07	0.06	0.06	0.07
glszm GrayLevelVariance	0.12	0.11	0.11	0.12	0.13	0.12	0.12	0.13	0.12	0.14	0.13	0.13	0.15
glszm HighGrayLevelZoneEmphasis	0.07	0.13	0.16	0.12	0.09	0.11	0.09	0.12	0.12	0.13	0.19	0.19	0.16
glszm LargeAreaEmphasis	0.49	2.66	1.06	0.91	0.59	0.52	0.43	0.69	0.51	0.45	1.51	1.26	1.07
glszm LargeAreaHighGrayLevelEmphasis	0.51	3.24	2.07	1.85	0.57	0.57	0.59	1.28	0.60	0.71	2.28	1.75	1.94
glszm LargeAreaLowGrayLevelEmphasis	0.38	0.67	0.42	0.37	0.50	0.44	0.49	0.38	0.32	0.27	0.51	0.35	0.40
glszm LowGrayLevelZoneEmphasis	0.27	0.17	0.27	0.21	0.23	0.23	0.23	0.29	0.31	0.21	0.29	0.31	0.26
glszm SizeZoneNonUniformity	0.45	0.27	0.28	0.26	0.47	0.47	0.43	0.25	0.24	0.24	0.30	0.25	0.28
glszm SizeZoneNonUniformityNormalized	0.14	0.07	0.11	0.09	0.17	0.17	0.19	0.10	0.11	0.08	0.11	0.09	0.10
glszm SmallAreaEmphasis	0.07	0.04	0.06	0.04	0.08	0.08	0.09	0.05	0.05	0.04	0.06	0.04	0.05
glszm SmallAreaHighGrayLevelEmphasis	0.10	0.13	0.11	0.13	0.14	0.16	0.15	0.12	0.11	0.12	0.13	0.16	0.12
glszm SmallAreaLowGrayLevelEmphasis	0.26	0.25	0.40	0.23	0.21	0.28	0.31	0.31	0.30	0.28	0.30	0.36	0.31
glszm ZoneEntropy	0.04	0.02	0.03	0.03	0.05	0.05	0.05	0.02	0.03	0.02	0.03	0.03	0.03
glszm ZonePercentage	0.21	0.19	0.11	0.13	0.25	0.23	0.24	0.10	0.09	0.08	0.14	0.10	0.12
glszm ZoneVariance	0.48	3.08	0.98	0.81	0.55	0.53	0.47	0.55	0.43	0.41	1.44	1.27	1.18
ngtdm Busyness	0.19	0.21	0.14	0.15	0.23	0.15	0.19	0.25	0.19	0.15	0.16	0.14	0.12
ngtdm Coarseness	0.07	0.06	0.05	0.04	0.12	0.12	0.13	0.14	0.10	0.08	0.04	0.04	0.04
ngtdm Complexity	0.14	0.15	0.11	0.11	0.14	0.17	0.14	0.18	0.13	0.12	0.17	0.11	0.11
ngtdm Contrast	0.92	0.25	0.47	0.39	0.34	0.22	0.19	0.12	0.09	0.12	0.89	0.46	0.21
ngtdm Strength	0.16	0.15	0.10	0.08	0.25	0.24	0.24	0.15	0.09	0.11	0.13	0.13	0.16

Table ST9: Median values of the relative difference between the values of the features computed in images with 2mm and 5mm slice thickness ($\Delta_r o$), across different non-extreme voxel size resampling and interpolation method, after correcting certain features for the dependence with the number of voxels. Values $\Delta_r \geq 0.5$ are shown in red.

Feature	O	(1,1,1)			$(\mu_x,\, \mu_y,\, Z)$			$(\mu_x,\, \mu_y,\, \mu_z)$			(1.25,1.25,1.25)		
		L	S	W	L	S	W	L	S	W	L	S	W
shape Elongation	0.18	0.20	0.20	0.20	0.20	0.20	0.20	0.18	0.18	0.18	0.18	0.18	0.18
shape Flatness	0.19	0.20	0.20	0.20	0.19	0.19	0.19	0.21	0.21	0.21	0.19	0.19	0.19
shape LeastAxisLength	0.14	0.12	0.12	0.12	0.13	0.13	0.13	0.12	0.12	0.12	0.12	0.12	0.12
shape MajorAxisLength	0.18	0.18	0.18	0.18	0.19	0.19	0.19	0.18	0.18	0.18	0.17	0.17	0.17
shape Maximum2DDiameterColumn	0.15	0.14	0.14	0.14	0.15	0.15	0.15	0.14	0.14	0.14	0.14	0.14	0.14
shape Maximum2DDiameterRow	0.14	0.14	0.14	0.14	0.14	0.14	0.14	0.14	0.14	0.14	0.14	0.14	0.14
shape Maximum2DDiameterSlice	0.10	0.09	0.09	0.09	0.08	0.08	0.08	0.11	0.11	0.11	0.08	0.08	0.08
shape Maximum3DDiameter	0.15	0.14	0.14	0.14	0.15	0.15	0.15	0.15	0.15	0.15	0.15	0.15	0.15
shape MeshVolume	0.21	0.21	0.21	0.21	0.21	0.21	0.21	0.21	0.21	0.21	0.20	0.20	0.20
shape MinorAxisLength	0.07	0.08	0.08	0.08	0.07	0.07	0.07	0.07	0.07	0.07	0.08	0.08	0.08
shape Sphericity	0.14	0.16	0.16	0.16	0.14	0.14	0.14	0.07	0.07	0.07	0.13	0.13	0.13
shape SurfaceArea	0.24	0.23	0.23	0.23	0.25	0.25	0.25	0.16	0.16	0.16	0.24	0.24	0.24
shape SurfaceVolumeRatio	0.14	0.14	0.14	0.14	0.13	0.13	0.13	0.06	0.06	0.06	0.12	0.12	0.12
shape VoxelVolume	0.20	0.21	0.21	0.21	0.20	0.20	0.20	0.21	0.21	0.21	0.20	0.20	0.20
firstorder 10Percentile	0.17	0.15	0.16	0.19	0.16	0.17	0.18	0.16	0.17	0.17	0.16	0.22	0.23
firstorder 90Percentile	0.04	0.04	0.04	0.05	0.04	0.05	0.05	0.05	0.05	0.06	0.04	0.05	0.05
firstorder Energy	0.19	0.17	0.17	0.18	0.21	0.20	0.20	0.22	0.21	0.20	0.20	0.15	0.15
firstorder Entropy	0.04	0.04	0.04	0.04	0.03	0.03	0.03	0.03	0.04	0.03	0.03	0.03	0.03
firstorder InterquartileRange	0.06	0.06	0.06	0.05	0.06	0.06	0.06	0.08	0.07	0.10	0.08	0.09	0.09
firstorder Kurtosis	0.10	0.13	0.10	0.10	0.10	0.09	0.09	0.15	0.12	0.12	0.18	0.14	0.11
firstorder Maximum	0.06	0.07	0.08	0.10	0.06	0.06	0.07	0.07	0.10	0.10	0.06	0.09	0.11
firstorder MeanAbsoluteDeviation	0.04	0.07	0.07	0.07	0.06	0.04	0.04	0.08	0.07	0.07	0.07	0.06	0.07
firstorder Mean	0.05	0.04	0.04	0.04	0.05	0.05	0.05	0.06	0.05	0.05	0.05	0.05	0.05
firstorder Median	0.04	0.04	0.03	0.04	0.04	0.05	0.05	0.05	0.05	0.06	0.04	0.04	0.04
firstorder Minimum	1.08	1.15	1.37	1.27	1.47	1.20	1.17	1.13	1.22	1.12	1.05	0.99	1.13
firstorder Range	0.11	0.18	0.17	0.20	0.13	0.11	0.10	0.14	0.11	0.11	0.17	0.16	0.14
firstorder RobustMeanAbsoluteDeviation	0.05	0.06	0.05	0.06	0.06	0.05	0.05	0.09	0.07	0.07	0.07	0.07	0.09
firstorder RootMeanSquared	0.05	0.05	0.05	0.05	0.05	0.05	0.05	0.05	0.05	0.05	0.06	0.05	0.06
firstorder Skewness	0.98	1.48	1.01	0.99	1.01	1.00	1.02	1.66	1.40	1.33	1.20	1.10	1.10
firstorder TotalEnergy	0.19	0.17	0.17	0.18	0.21	0.20	0.20	0.22	0.21	0.20	0.20	0.15	0.15
firstorder Uniformity	0.12	0.12	0.14	0.13	0.11	0.12	0.13	0.13	0.14	0.11	0.13	0.12	0.10
firstorder Variance	0.11	0.17	0.14	0.15	0.13	0.11	0.11	0.20	0.15	0.16	0.18	0.14	0.15
glcm Autocorrelation	0.22	0.22	0.17	0.19	0.19	0.18	0.19	0.24	0.22	0.21	0.30	0.23	0.21
glcm ClusterProminence	0.33	0.39	0.41	0.47	0.23	0.35	0.37	0.38	0.42	0.43	0.34	0.40	0.39
glcm ClusterShade	1.02	1.36	1.08	1.40	1.01	1.00	1.02	1.29	1.19	1.32	1.45	1.30	1.33
glcm ClusterTendency	0.20	0.17	0.23	0.26	0.13	0.19	0.20	0.28	0.29	0.26	0.23	0.22	0.19
glcm Contrast	0.26	0.31	0.26	0.31	0.30	0.26	0.30	0.25	0.25	0.20	0.31	0.23	0.23
glcm Correlation	0.19	0.15	0.17	0.19	0.20	0.17	0.17	0.15	0.20	0.20	0.22	0.22	0.27
glcm DifferenceAverage	0.15	0.16	0.14	0.16	0.13	0.13	0.13	0.13	0.12	0.12	0.16	0.12	0.11
glcm DifferenceEntropy	0.05	0.06	0.05	0.06	0.04	0.04	0.05	0.04	0.04	0.04	0.06	0.04	0.04
glcm DifferenceVariance	0.26	0.29	0.24	0.28	0.26	0.29	0.30	0.24	0.17	0.22	0.31	0.23	0.24
glcm Id	0.08	0.10	0.08	0.10	0.08	0.08	0.07	0.08	0.08	0.06	0.10	0.07	0.07
glcm Idm	0.11	0.15	0.11	0.14	0.12	0.11	0.11	0.12	0.11	0.09	0.14	0.10	0.10
glcm Idmn	0.01	0.01	0.01	0.01	0.01	0.01	0.01	0.01	0.01	0.01	0.01	0.01	0.01
glcm Idn	0.02	0.01	0.02	0.02	0.01	0.01	0.01	0.01	0.01	0.01	0.02	0.01	0.01
glcm Imc1	0.16	0.12	0.12	0.13	0.16	0.16	0.17	0.20	0.19	0.19	0.13	0.17	0.18
glcm Imc2	0.05	0.05	0.04	0.04	0.06	0.05	0.06	0.06	0.07	0.06	0.05	0.07	0.07
glcm InverseVariance	0.13	0.14	0.12	0.14	0.10	0.10	0.11	0.13	0.09	0.10	0.13	0.11	0.08
glcm JointAverage	0.11	0.12	0.09	0.10	0.10	0.08	0.10	0.12	0.10	0.11	0.14	0.12	0.10
glcm JointEnergy	0.23	0.30	0.27	0.22	0.26	0.26	0.25	0.23	0.23	0.20	0.26	0.19	0.22
glcm JointEntropy	0.05	0.04	0.04	0.04	0.04	0.05	0.05	0.03	0.03	0.03	0.04	0.03	0.03
glcm MCC	0.13	0.12	0.11	0.11	0.15	0.15	0.15	0.13	0.14	0.11	0.13	0.16	0.15
glcm MaximumProbability	0.26	0.26	0.20	0.23	0.22	0.24	0.23	0.21	0.18	0.19	0.25	0.19	0.24
glcm SumAverage	0.11	0.12	0.09	0.10	0.10	0.08	0.10	0.12	0.10	0.11	0.14	0.12	0.10
glcm SumEntropy	0.03	0.03	0.04	0.03	0.03	0.03	0.03	0.03	0.04	0.04	0.03	0.03	0.03
glcm SumSquares	0.22	0.21	0.26	0.28	0.19	0.23	0.26	0.22	0.25	0.24	0.22	0.22	0.23

Table ST10: Median values of the relative difference between the values of the feature ratios (tumour over muscle) computed in images with 2mm and 5mm slice thickness ($\Delta_{\rho,r}$), across different non-extreme voxel size resampling and interpolation method. Values $\Delta_{\rho,r} \geq 0.5$ are shown in red.

Feature	O	(1,1,1)			(μ_x, μ_y, Z)			(μ_x, μ_y, μ_z)			(1.25,1.25,1.25)		
		L	S	W	L	S	W	L	S	W	L	S	W
gldm DependenceEntropy	0.02	0.02	0.01	0.01	0.02	0.01	0.01	0.02	0.01	0.02	0.02	0.01	0.02
gldm DependenceNonUniformity	0.17	0.15	0.11	0.12	0.19	0.16	0.17	0.15	0.15	0.16	0.16	0.16	0.14
gldm DependenceNonUniformityNormalized	0.09	0.12	0.10	0.11	0.12	0.11	0.10	0.10	0.11	0.09	0.13	0.09	0.10
gldm DependenceVariance	0.20	0.16	0.20	0.20	0.21	0.21	0.17	0.23	0.15	0.14	0.27	0.19	0.16
gldm GrayLevelNonUniformity	0.19	0.20	0.23	0.21	0.16	0.19	0.20	0.29	0.26	0.26	0.18	0.22	0.21
gldm GrayLevelVariance	0.22	0.20	0.23	0.26	0.19	0.23	0.24	0.20	0.22	0.23	0.21	0.20	0.20
gldm HighGrayLevelEmphasis	0.20	0.18	0.17	0.17	0.15	0.15	0.16	0.21	0.18	0.20	0.29	0.20	0.18
gldm LargeDependenceEmphasis	0.15	0.17	0.17	0.17	0.19	0.17	0.19	0.15	0.19	0.13	0.21	0.15	0.14
gldm LargeDependenceHighGrayLevelEmphasis	0.26	0.37	0.27	0.22	0.28	0.18	0.21	0.27	0.26	0.26	0.30	0.25	0.25
gldm LargeDependenceLowGrayLevelEmphasis	0.32	0.31	0.33	0.32	0.26	0.29	0.28	0.37	0.36	0.34	0.32	0.35	0.27
gldm LowGrayLevelEmphasis	0.37	0.34	0.28	0.38	0.38	0.30	0.27	0.36	0.38	0.37	0.34	0.47	0.40
gldm SmallDependenceEmphasis	0.10	0.15	0.12	0.12	0.12	0.12	0.12	0.10	0.14	0.09	0.17	0.10	0.10
gldm SmallDependenceHighGrayLevelEmphasis	0.17	0.20	0.26	0.27	0.17	0.18	0.18	0.17	0.19	0.23	0.21	0.19	0.24
gldm SmallDependenceLowGrayLevelEmphasis	0.39	0.37	0.39	0.39	0.43	0.34	0.37	0.48	0.40	0.36	0.36	0.50	0.46
glrlm GrayLevelNonUniformity	0.18	0.18	0.21	0.19	0.14	0.18	0.18	0.29	0.26	0.25	0.19	0.22	0.22
glrlm GrayLevelNonUniformityNormalized	0.12	0.12	0.13	0.13	0.12	0.12	0.12	0.13	0.14	0.11	0.13	0.12	0.10
glrlm GrayLevelVariance	0.22	0.21	0.23	0.26	0.18	0.23	0.24	0.20	0.22	0.23	0.21	0.20	0.21
glrlm HighGrayLevelRunEmphasis	0.20	0.18	0.16	0.17	0.15	0.15	0.17	0.21	0.18	0.20	0.29	0.20	0.18
glrlm LongRunEmphasis	0.02	0.04	0.03	0.03	0.03	0.02	0.02	0.02	0.02	0.02	0.04	0.02	0.02
glrlm LongRunHighGrayLevelEmphasis	0.22	0.27	0.17	0.21	0.17	0.15	0.17	0.24	0.19	0.20	0.30	0.22	0.19
glrlm LongRunLowGrayLevelEmphasis	0.37	0.31	0.23	0.36	0.39	0.29	0.27	0.38	0.37	0.36	0.35	0.41	0.38
glrlm LowGrayLevelRunEmphasis	0.37	0.34	0.28	0.36	0.38	0.30	0.26	0.38	0.37	0.37	0.33	0.46	0.38
glrlm RunEntropy	0.03	0.02	0.03	0.03	0.02	0.03	0.03	0.03	0.03	0.03	0.02	0.03	0.02
glrlm RunLengthNonUniformity	0.12	0.13	0.11	0.11	0.13	0.13	0.13	0.14	0.16	0.17	0.17	0.17	0.16
glrlm RunLengthNonUniformityNormalized	0.01	0.02	0.02	0.02	0.01	0.01	0.01	0.01	0.01	0.01	0.02	0.01	0.01
glrlm RunPercentage	0.01	0.01	0.01	0.01	0.01	0.01	0.01	0.01	0.01	0.01	0.01	0.01	0.01
glrlm RunVariance	0.14	0.21	0.19	0.18	0.18	0.19	0.16	0.13	0.19	0.18	0.25	0.17	0.15
glrlm ShortRunEmphasis	0.01	0.01	0.01	0.01	0.01	0.00	0.01	0.00	0.01	0.00	0.01	0.01	0.01
glrlm ShortRunHighGrayLevelEmphasis	0.20	0.18	0.17	0.16	0.15	0.15	0.16	0.19	0.18	0.19	0.28	0.21	0.18
glrlm ShortRunLowGrayLevelEmphasis	0.37	0.34	0.29	0.35	0.39	0.30	0.26	0.37	0.37	0.37	0.34	0.46	0.38
glszm GrayLevelNonUniformity	0.16	0.17	0.11	0.17	0.19	0.15	0.15	0.17	0.16	0.14	0.19	0.20	0.17
glszm GrayLevelNonUniformityNormalized	0.11	0.09	0.11	0.11	0.08	0.10	0.10	0.09	0.11	0.10	0.09	0.11	0.09
glszm GrayLevelVariance	0.20	0.19	0.19	0.19	0.15	0.18	0.18	0.14	0.19	0.20	0.13	0.19	0.16
glszm HighGrayLevelZoneEmphasis	0.18	0.16	0.15	0.16	0.15	0.16	0.16	0.20	0.17	0.21	0.24	0.20	0.18
glszm LargeAreaEmphasis	0.32	0.45	0.38	0.38	0.36	0.26	0.31	0.31	0.38	0.32	0.58	0.30	0.38
glszm LargeAreaHighGrayLevelEmphasis	0.42	0.81	0.48	0.43	0.47	0.40	0.36	0.42	0.46	0.34	0.57	0.36	0.47
glszm LargeAreaLowGrayLevelEmphasis	0.46	0.44	0.38	0.49	0.28	0.31	0.36	0.46	0.55	0.41	0.47	0.28	0.37
glszm LowGrayLevelZoneEmphasis	0.37	0.28	0.32	0.38	0.42	0.29	0.29	0.36	0.32	0.37	0.35	0.49	0.37
glszm SizeZoneNonUniformity	0.22	0.27	0.23	0.21	0.24	0.22	0.22	0.23	0.26	0.20	0.27	0.22	0.23
glszm SizeZoneNonUniformityNormalized	0.08	0.10	0.11	0.09	0.12	0.10	0.10	0.10	0.10	0.09	0.12	0.08	0.10
glszm SmallAreaEmphasis	0.04	0.05	0.06	0.05	0.06	0.05	0.05	0.05	0.05	0.05	0.06	0.04	0.05
glszm SmallAreaHighGrayLevelEmphasis	0.17	0.17	0.18	0.20	0.13	0.14	0.15	0.16	0.15	0.18	0.22	0.19	0.24
glszm SmallAreaLowGrayLevelEmphasis	0.39	0.35	0.34	0.36	0.43	0.29	0.32	0.42	0.33	0.33	0.34	0.53	0.51
glszm ZoneEntropy	0.02	0.01	0.01	0.01	0.02	0.02	0.02	0.01	0.02	0.01	0.02	0.02	0.02
glszm ZonePercentage	0.09	0.15	0.13	0.13	0.12	0.10	0.12	0.09	0.12	0.10	0.17	0.10	0.09
glszm ZoneVariance	0.45	0.66	0.51	0.56	0.53	0.45	0.45	0.50	0.45	0.43	0.74	0.40	0.54
ngtdm Busyness	0.17	0.26	0.19	0.22	0.20	0.22	0.19	0.19	0.19	0.18	0.22	0.16	0.19
ngtdm Coarseness	0.13	0.14	0.14	0.14	0.16	0.13	0.12	0.18	0.16	0.17	0.18	0.19	0.18
ngtdm Complexity	0.19	0.16	0.18	0.20	0.16	0.18	0.18	0.21	0.16	0.16	0.20	0.17	0.18
ngtdm Contrast	0.30	0.36	0.34	0.36	0.24	0.22	0.26	0.25	0.28	0.26	0.29	0.30	0.29
ngtdm Strength	0.20	0.22	0.22	0.21	0.18	0.16	0.14	0.13	0.17	0.16	0.18	0.15	0.20

Table ST11: Median values of the relative difference between the values of the feature ratios (tumour over muscle) computed in images with 2mm and 5mm slice thickness ($\Delta_{\rho,r}$), across different non-extreme voxel size resampling and interpolation method. Values $\Delta_{\rho,r} \geq 0.5$ are shown in red.
